# Nonlinear Finite Element Model for Bending Analysis of Functionally-Graded Porous Circular/Annular Micro-Plates under Thermomechanical Loads Using Quasi-3D Reddy Third-Order Plate Theory

**DOI:** 10.3390/ma16093505

**Published:** 2023-05-02

**Authors:** Jinseok Kim, Enrique Nava, Semsi Rakici

**Affiliations:** 1 Department of Mechanical and Aerospace Engineering, Western Michigan University, Kalamazoo, MI 49008, USA; enrique.navamunoz@wmich.edu; 2 School of Building Construction, Georgia Institute of Technology, Atlanta, GA 30332, USA

**Keywords:** nonlinear finite element analysis, axisymmetric plates, quasi-3D Reddy third-order theory, functionally-graded porous materials, modified couple stress theory

## Abstract

A nonlinear finite element model for axisymmetric bending of micro circular/annular plates under thermal and mechanical loading was developed using quasi-3D Reddy third-order shear deformation theory. The developed finite element model accounts for a variation of material constituents utilizing a power-law distribution of a two-constituent material, three different porosity distributions through plate thickness, and geometrical nonlinearity. The modified couple stress theory was utilized to account for the strain gradient effects using a single material length scale parameter. Three different types of porosity distributions that have the same overall volume fraction but different enhanced areas were considered as a form of cosine functions. The effects of the material and porosity distribution, microstructure-dependency, the geometric nonlinearity, and various boundary conditions on the bending response of functionally-graded porous axisymmetric microplates under thermomechanical loads were studied using the developed nonlinear finite element model.

## 1. Introduction

Functionally-graded materials (FGMs) are advanced engineering materials composed of two or more constituents with a continuous variation in their compositions. Unlike FGMs, laminated composites exhibit immediate changes in thermal and mechanical properties of the constituents, resulting in stress concentrations at the interfaces where two discrete materials bond together. This leads to delamination problems and the presence of residual stresses in conventional composites working under severe conditions. FGMs were developed by researchers in Japan in 1984 to overcome these issues encountered in a thermal coating material requirement of a hypersonic space plane project [1]. Since then, FGMs have been used in various fields such as aerospace, automobile, electronic, and medical industries due to their advantages over laminated composites and their flexibility to be designed according to the needs of the application field and working environment. The reader is referred to the following review articles [2,3,4,5] for details of the historical development of these materials, manufacturing techniques, and optimization of their functionality.

The FGMs have great potential for improving the performance of various components in engineering structures, especially circular and annular plates. Over the last few decades, researchers have extensively studied the behavior of functionally-graded (FG) circular and annular plates under thermal, mechanical, and combined thermomechanical loadings. Since FGMs were initially designed to withstand extreme thermal environments, most of the literature focuses on their thermal analysis. Typical FGMs used in these studies are made from a mixture of ceramics for their low thermal conductivity and metals for their ductility and resistance to fracture caused by stresses likely to occur in high-temperature gradients. Additionally, the majority of studies on FG plates employ a power law or exponential distribution of materials through the thickness direction of the plates

In 1998, Reddy and Chin [6] conducted a numerical study to investigate thermomechanical responses of FG cylinders and plates under extreme thermal loading conditions using the first-order shear deformation plate theory (FSDT). In their study, the effects of thermomechanical coupling on the response of FGMs subjected to thermal shock were investigated. For the functionally-graded axisymmetric cylinder subjected to high thermal loading, the temperature distribution obtained from both coupled and uncoupled formulations did not show significant differences. However, it was observed that the radial stresses were more affected than the hoop stresses in the FG cylinder.

Using the FSDT, exact solutions of the static bending analysis of FG circular and annular plates having various boundary conditions were presented by Reddy et al. [7]. They derived the solutions of deflections, forces, and the moments of the FG plates based on FSDT in terms of the associated quantities for the isotropic plates based on the classical plate theory (CPT). Hence, the bending solutions of the FG circular plate became readily available whenever the CPT solution was known. Ma and Wang [8] studied the axisymmetric nonlinear bending and post buckling response of functionally-graded circular plates under thermal, mechanical and combined thermomechanical loading conditions. In this study, governing equations were derived using the von Kármán plate theory and the numerical solutions were obtained with the help of the shooting method. The results of this study showed that temperature distribution, deflection values, critical buckling temperature, and post buckling behavior of the functionally-graded circular plates were significantly affected by the volume fraction index.

Praveen and Reddy [9] introduced the finite element formulations that account for the transverse shear strains, rotary inertia, and von Kármán nonlinear strains to perform static and dynamic thermoelastic analysis of the functionally-graded ceramic-metal plates based on FSDT. In 2000, Reddy [10] presented the formulation and analytical solution of simply-supported rectangular FG plates using third order shear deformation plate theory (TSDT) including thermomechanical coupling, time dependency, and von Kármán geometric nonlinearity. From these two studies, it was concluded that the distribution of material constituents in the functionally-graded plates had a significant influence on the resulting thermoelastic response of FG plates. Najafizadeh and Heydari [11] investigated the thermal buckling analysis of functionally-graded circular plates under both uniform and non-uniform temperature changes by employing the TSDT.

Prakash and Ganapathi [12] employed the finite element method to carry out asymmetric-free vibration and thermoelastic stability analysis of functionally-graded circular plates. Nie and Zhong [13] studied the three-dimensional free and forced vibration analysis of functionally-graded circular plates and found that the lowest nondimensional frequency and circumferential wave number of the plate increased as the thickness-to-width ratio increased. They also observed that the magnitudes of the displacements and stresses became larger as the forcing frequency approached the natural frequency of the FG circular plate.

Efraim and Eisenberger [14] presented the free vibration analysis of variable thickness thick annular plates using the exact element method and the dynamic stiffness method. They used FSDT in their formulations and varied Poisson ratio according to the power law distribution in addition to elastic modulus and mass density. Golmakani and Kadkhodayan [15] presented another study that accounted for the gradation of Poisson ratio. They investigated the nonlinear bending analysis of annular FG plates based on both FSDT and TSDT. The same authors [16] later performed a large deflection analysis of circular and annular FG plates subjected to thermomechanical loading within the framework of FSDT, including von Kármán nonlinearity.

Saidi et al. [17] employed unconstrained third order shear deformation theory to analyze the axisymmetric bending and buckling behavior of thick FG circular plates.

Nosier and Fallah [18] reformulated governing equations of the FSDT into interior and edge-zone equations for functionally-graded circular plates. By introducing two sets of equations to define the edge-zone problem, they uncoupled the bending and extension equations, which made it possible to obtain analytical solutions for the asymmetric behavior of functionally-graded circular plates with various boundary conditions under mechanical and thermal loading. Later, they included the von Kármán nonlinear strains into their formulations and investigated the axisymmetric and asymmetric nonlinear bending of functionally-graded circular plates subject to linearly-varying transverse loading [19]. The axisymmetric bending analysis of FG circular plates under arbitrary transverse loads was studied by Yun et al. [20]. They obtained the analytical solutions for the FG circular plates with elastic simple and rigid slipping supports cases when the material property of the FG plate was varying with an exponential distribution. Another analytical study was conducted to solve for in-plane and out-of-plane free vibrations of thick FG circular and annular plates embedded in piezoelectric layers by Talabi and Saidi [21], employing TSDT. The effects of both electrical and mechanical boundary conditions, geometrical parameters of the plate, and in-plane displacements on the middle plane on the natural frequencies of FG circular and annular plates were discussed. Żur [22] applied the Neumann series method to investigate the free vibration behavior of discrete-continuous FG circular plates that may have several ring attachments such as masses, springs and damping elements.

The FG circular and annular plates can be further improved by adding porosity into their composition to decrease the weight of the structure and/or increase the insulation properties. Hence, it is important to examine the mechanical and thermal responses of FG porous plates under different loading and boundary conditions. A general solution of a porous FG circular plate that is supported by a non-uniform Kerr elastic foundation and subjected to non-axisymmetric, non-uniform shear and normal tractions, and a magnetic actuation was developed by Rad and Shariyat [23]. Their results showed that the radial displacement component was more prone to being affected by the induced magnetic actuation. Additionally, because of the presence of incompressible fluid in the pores in this study, as the porosity increased, the plates became stiffer. The buckling behavior of porous circular plate between piezoelectric layers under thermal loading was investigated by Jabbari et al. [24]. They showed that, as the porosity increased, the critical temperature decreased and the plate whose pores were saturated with fluid became unstable. On the other hand, the critical temperature of the plates can be decreased by increasing the thermal expansion coefficient of the fluid filling the pores and the piezoelectric layers. Zhao et al. [25] studied the free and forced vibration analysis of FG porous circular, annular, and sector plates with general elastic restraints using FSDT.

These extensive studies conducted on FG circular and annular plates show that these structures have an intrinsic advantage resulting from the non-homogeneity and smooth variations of the material properties. It is shown that the deflections and tensile stresses of FG circular and annular plates can be lower and critical buckling loads can be higher as compared to the homogeneous ones, depending on the predetermined variation of material properties of FG circular and annular plates. It is also possible to adjust the natural frequencies of these structures by changing the variation of the material distribution. Hence, all these conclusions make it attractive to examine the performance of the FGMs for the micro-scale structures. However, conventional continuum mechanics cannot capture the size dependency that is experimentally observed at the micro-scale [26,27,28,29]. Therefore, a higher order continuum theory is required for the accurate modeling and analysis of these structures. Couple stress theories [30,31,32], Erigen nonlocal elasticity theory [33] and the strain gradient elasticity theories [26,34,35] are some of the higher order continuum theories that take the size dependency into account. The modified couple stress theory is the most commonly employed theory because only a single length scale parameter is needed to include size effect.

Ke et al. [36] investigated the bending, buckling, and free vibration analyses of FG annular microplates with hinged–hinged and clamped–clamped boundary conditions. Their size-dependent annular microplate model was based on the Mindlin plate theory and the modified couple stress theory. This study showed that elastic buckling analysis was more sensitive to size effect than the free vibration analysis. Similar analyses were presented by Ansari et al. [37] for FG circular and annular microplates. They also employed Mindlin plate theory, but different to the previous study, size dependency was included using modified strain gradient elasticity theory. Both studies agreed that the smaller the dimensionless length scale parameter they had, the smaller the deflection but the higher the critical buckling load and natural frequencies that they obtained.

Reddy and Berry [38] presented the classical and the first order plate theories for axisymmetric bending of circular micro-plates including von Kármán nonlinear strains. Size dependency was captured with the modified couple stress theory. Later, Reddy et al. [39] used this theory to develop nonlinear finite element models for FG circular plates.

An analytical solution for the free vibration of FG circular and annular nanoplates was obtained by Hosseini-Hashemi et al. [40] based on Mindlin plate theory and Eringen nonlocal elasticity theory. Beni et al. [41] studied the same problem for FG cylindrical nanoshells using FSDT in conjunction with the modified couple stress theory. They presented the effects of the material length scale, distribution of the FGMs, nanotube thickness, and length on the fundamental frequencies. Eshraghi et al. [42] studied the bending and the free vibration analysis of FG annular and circular microplates subjected to thermal loading using the modified couple stress theory. They unified the displacement fields such that results for Kirchoff plate theory, Mindlin plate theory, and third order shear deformation plate theory can be generated. Additionally, not only the mechanical and thermal properties of the FG plates but also the material length scale parameter were not kept constant but were changed through the thickness direction, obeying a power law distribution. The transverse deflections, normalized circumferential and radial stresses, and the natural frequencies were presented for different thermal loading, material, and geometrical parameters. Ji et al. [43] developed a plate model capturing the size dependency for FG circular micro-plates based on the strain gradient theory of Zhou. They analyzed the bending and free vibration of a simply-supported circular micro-plate and the results were compared with those obtained by employing the strain gradient theory of Lam, the modified couple stress theory, and the CPT.

A free vibration and thermal buckling analysis of an FG porous circular micro-plate was conducted by Shojaeefard et al. [44] based on CPT and FSDT with modified couple stress theory. The effects of the temperature change, distribution of the material properties, size-dependency, and porosity on the fundamental frequencies and critical temperature were investigated. Kim et al. [45] presented the analytical solutions of bending, free vibration, and the buckling problem for FG porous micro-plates using CPT and FSDT in conjunction with the modified couple stress theory. Recently, Wang and Zhang [46] studied the thermal buckling and postbuckling responses of GPL-reinforced nanocomposite beams using the higher order shear deformation theory with temperature-dependent properties. Zhang et al. [47] carried out analytical studies on thermo-mechanical responses of porous functionally-graded, graphene-reinforced cylindrical panels based on a third order shear deformation theory. The acoustic characteristics of functionally-graded porous graphene reinforced nano composite plates (FG-PGRC) were studied by Xu et al. [48]. In their study, a higher order shear deformation theory was utilized to study the vibration and noise reduction of an FG-PGRC plate.

This study aimed to investigate the behavior of FG porous circular microplates under thermal and mechanical loadings, which has not been studied in the literature. To this end, a nonlinear finite element model was developed based on quasi-3D Reddy third-order shear deformation theory and the modified couple stress theory, taking into account von Kármán nonlinear strains to consider geometrical nonlinearity. The FGM was composed of two constituents based on a power law distribution through the thickness direction, and three different porosity profiles were considered. Parametric analyses were conducted to investigate the effects of the distribution of material properties and porosity, size-dependency, geometric nonlinearity, and different boundary conditions on the static bending analysis of FG porous circular microplates.

## 2. Constitutive Models

### 2.1. Functionally-Graded Porous Materials

**Figure 1:**
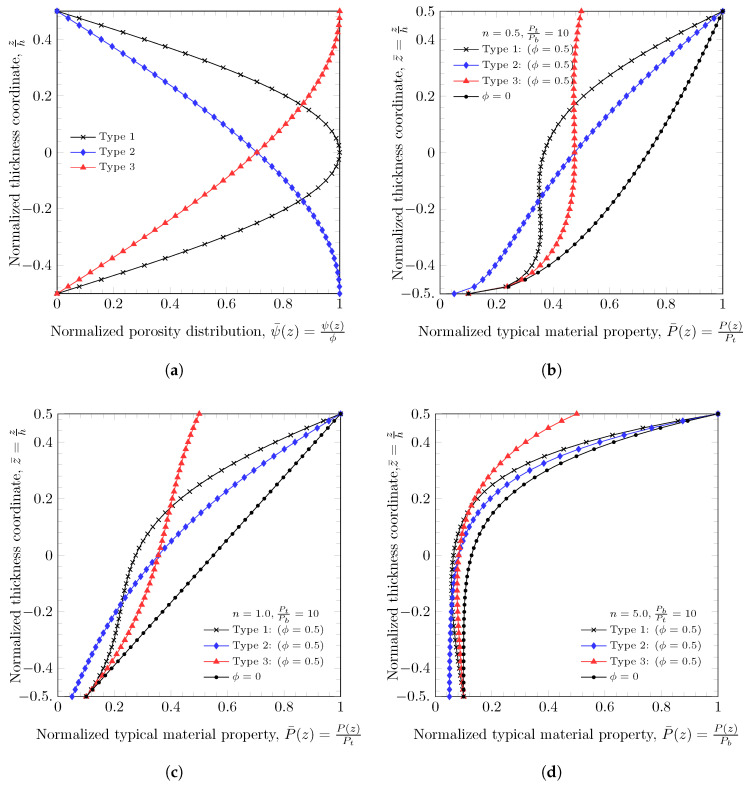
Distribution of porosity and a typical material property [45]. (**a**) Distribution of porosity through thickness. (**b**) Distribution of typical material property (n=0.5). (**c**) Distribution of typical material property (n=1.0). (**d**) Distribution of typical material property (n=5.0).

The model considers isotropic axisymmetric plates composed of two constituents with varying material properties and internal porosity through the thickness, modeled using a power-law distribution and cosine variation, respectively. The typical material properties of functionally-graded porous materials (FGPM) are thus captured in the model, as shown in Equation (1).

(1) $$P\left(z\right)=\left[\left(P_{t}-P_{b}\right)f\left(z\right)+P_{b}\right]\left(1-\psi (z)\right),\;f\left(z\right)={\left(\frac{z}{h}+\frac{1}{2}\right)}^{n},$$

where $P_{t}$ and $P_{b}$ are material properties on the top and bottom surfaces of plates, *n* is power-index, f(z) is a volume fraction function, and ψ(z) is a porosity distribution function. Three different types of porosity distributions were considered in this study.

(2) $$\begin{array}{cc} \mathrm{Type}\;1:\psi (z) & =\phi \;\cos\left[\pi \left(\frac{z}{h}\right)\right]\\ \mathrm{Type}\;2:\psi (z) & =\phi \;\cos\left[\frac{\pi }{2}\left(\frac{z}{h}+0.5\right)\right]\\ \mathrm{Type}\;3:\psi (z) & =\phi \;\cos\left[\frac{\pi }{2}\left(\frac{z}{h}-0.5\right)\right], \end{array}$$

where ϕ is the maximum porosity value along thickness direction. The distribution of porosity through the thickness of the plates was normalized to have the same porous volume, and it is important to investigate the effect of different porosity distributions [45]. Figure 1a displays the normalized porosity distribution throughout the thickness of the plate. Figure 1b–d show the effects of porosity distributions on the variation of typical materials properties. As an example, a porosity value was set to ϕ=0.5, three different power-law index values n=0,0.5, and 5.0 were set. The ratio of material properties on the top and bottom surfaces was assumed to be $\frac{E_{t}}{E_{b}}=10$. The Type 1, Type 2, and Type 3 porosity distributions are a symmetric and center-enhanced, a bottom area enhanced, and a top area enhanced porosity distributions, respectively.

### 2.2. Modified Couple Stress Theory

The motion of the material particles in classical couple stress theory [30,49] is described to rotate the material particles in addition to forces in the classical continuum mechanics. The size-dependent effect was captured using two additional material constants in the classical couple stress theory. These two material constants are difficult to determine because of their indeterminacy. Eringen [33] proposed a micropolar theory and defined the motion of a particle using the location vector and inner product of a rigid vector. A modified couple stress theory using the concept of the representative volume elements and a higher order equilibrium condition was proposed by Yang et al. [32]. According to the modified couple stress theory, the deviatoric part of a couple stress tensor is only associated with the symmetric part of rotation gradient and it contributes to the total strain energy along with the classical strain energy. The strain energy potential of an axisymmetric plate based on the modified couple stress theory can be expressed as

(3) $$\begin{array}{cc} U & =\frac{1}{2}\int_{V}\left(\sigma :\varepsilon +m:\chi \right)dV\\ \; & =\frac{1}{2}\int_{r_{i}}^{r_{o}}\left[\int_{-\frac{h}{2}}^{\frac{h}{2}}\left(\sigma :\varepsilon +m:\chi \right)dz\right]rdr, \end{array}$$

where $r_{i}$ and $r_{o}$ are the inner and outer radii of the plate, σ and ε are the Cauchy stress tensor and Von Kámán nonlinear strain tensor, m and χ are the deviatoric part of the symmetric couple stress tensor and the symmetric curvature tensor. Note that the differential volume element dV can be written as dV=rdrdθdz and 2π from the integration with respect to θ being omitted in Equation (3). The curvature tensor and the deviatoric part of the symmetric couple stress tensor are defined as [32]

(4) $$\begin{array}{cc} \chi & =\frac{1}{2}\left[\nabla \omega +{\left(\nabla \omega \right)}^{T}\right] \end{array}$$

(5) $$\begin{array}{cc} m & =2\mu \ell^{2}\chi , \end{array}$$

where ω is the rotation vector, $\omega =\frac{1}{2}\nabla \times u$, μ is the shear modulus, and *ℓ* is a length scale parameter.

In this study, an isotropic linear elastic material was assumed and the stress and strain relation [50] for an axisymmetric plate is

(6) $$\left\{\begin{array}{c} \sigma_{rr}\\ \sigma_{\theta \theta }\\ \sigma_{zz}\\ \sigma_{rz} \end{array}\right\}=\Lambda \left[\begin{array}{cccc} 1-\nu & \nu & \nu & 0\\ \nu & 1-\nu & \nu & 0\\ \nu & \nu & 1-\nu & 0\\ 0 & 0 & 0 & \frac{1}{2}\left(1-2\nu \right) \end{array}\right]\left\{\begin{array}{c} \varepsilon_{rr}-\alpha \left(T-T_{0}\right)\\ \varepsilon_{\theta \theta }-\alpha \left(T-T_{0}\right)\\ \varepsilon_{zz}-\alpha \left(T-T_{0}\right)\\ \varepsilon_{rz} \end{array}\right\},$$

where Λ=E/[(1+ν)(1−2ν)], *E* is Young’s modulus, which varies along the plate’s thickness, ν is a constant Poisson’s ratio in the elastic stiffness matrix. α is the thermal expansion coefficient, and *T* and $T_{0}$ are the temperature at a material point and the reference temperature of the undeformed body.

The nonzero curvatures and modified couple stresses are

(7) $$\left\{\begin{array}{c} m_{r\theta }\\ m_{\theta z} \end{array}\right\}=2\ell^{2}\mu \left\{\begin{array}{c} \chi_{r\theta }\\ \chi_{\theta z} \end{array}\right\}.$$

## 3. Quasi-3D Reddy Third-Order Plate Theory

### 3.1. Displacement and Strains

The displacement field of quasi-3D Reddy third-order plate theory can be derived from an assumption of a cubic variation of in-plane displacements and a quadratic variation of deflection (i.e., out-of-plane displacement) with zero tangential traction on top and bottom surfaces. The displacement field of cubic variation of in-plane displacement and a quadratic variation of deflection through thickness direction for axisymmetric plates takes the form of

(8) $$\begin{array}{cc} u_{r}\left(r,z,t\right) & =u_{0}\left(r,t\right)+z\theta_{r}\left(r,t\right)+z^{2}\phi_{r}\left(r,t\right)+z^{3}\psi_{r}\left(r,t\right)\\ u_{z}\left(r,z,t\right) & =w_{0}\left(r,t\right)+z\theta_{z}\left(r,t\right)+z^{2}\phi_{z}\left(r,t\right). \end{array}$$

With the assumption of zero tangential traction on top and bottom surfaces, the displacement (8) can be written in the form of

(9) $$\varepsilon_{rz}\left(r,\frac{h}{2},t\right)=\varepsilon_{rz}\left(r,-\frac{h}{2},t\right)=0.$$

The form of quasi-3D Reddy third-order plate theory for axisymmetric plates takes

(10) $$\begin{array}{cc} u_{r}\left(r,z,t\right) & =u_{0}\left(r,t\right)+z\theta_{r}\left(r,t\right)-\frac{z^{2}}{2}\frac{\partial \theta_{z}}{\partial r}-z^{3}c_{1}\left[\theta_{r}\left(r,t\right)+\frac{\partial \lambda \left(r,t\right)}{\partial r}\right]\\ u_{z}\left(r,z,t\right) & =w_{0}\left(r,t\right)+z\theta_{z}\left(r,t\right)+z^{2}\phi_{z}\left(r,t\right), \end{array}$$

where $u_{0}$ is the membrane displacement, $\theta_{r}$ is the rotation of a transverse normal about θ direction, $w_{0}$ is the deflection, $\theta_{z}$ and $\phi_{z}$ are the thickness stretch, $\lambda =w_{0}+\frac{h^{2}}{4}\phi_{z}$ and $c_{1}=\frac{4}{3h^{2}}$.

Based on the assumption of small strains and moderate rotations, nonzero von Kámán nonlinear strain for the axisymmetric plate is given by [39].

(11) $$\begin{array}{cc} \varepsilon_{rr} & =\frac{\partial u_{r}}{\partial r}+\frac{1}{2}{\left(\frac{\partial u_{z}}{\partial r}\right)}^{2}\\ \varepsilon_{\theta \theta } & =\frac{u_{r}}{r}\\ \varepsilon_{zz} & =\frac{\partial u_{z}}{\partial z}\\ \varepsilon_{rz} & =\frac{1}{2}\left(\frac{\partial u_{r}}{\partial z}+\frac{\partial u_{z}}{\partial r}\right). \end{array}$$

The non-zero strains with the displacement field (10) of quasi-3D Reddy third-order plate theory are defined as

(12) $$\begin{array}{cc} \left\{\begin{array}{c} \varepsilon_{rr}\\ \varepsilon_{\theta \theta }\\ \varepsilon_{zz}\\ 2\varepsilon_{rz} \end{array}\right\}\; & =\left\{\begin{array}{c} \frac{\partial u_{0}}{\partial r}+\frac{1}{2}{\left(\frac{\partial w_{0}}{\partial r}\right)}^{2}\\ \frac{u_{0}}{r}\\ \theta_{z}\\ \theta_{r}+\frac{\partial w_{0}}{\partial r} \end{array}\right\}+z\left\{\begin{array}{c} \frac{\partial \theta_{r}}{\partial r}\\ \frac{\theta_{r}}{r}\\ 2\phi_{z}\\ 0 \end{array}\right\}-z^{2}\left\{\begin{array}{c} \frac{1}{2}\frac{\partial^{2}\theta_{z}}{\partial r^{2}}\\ \frac{1}{2r}\frac{\partial \theta_{z}}{\partial r}\\ 0\\ -c_{2}\left(\theta_{r}+\frac{\partial \lambda }{\partial r}\right)+\frac{\partial \phi_{z}}{\partial r} \end{array}\right\}\\ \; & \;-z^{3}\left\{\begin{array}{c} c_{1}\left(\frac{\partial \theta_{r}}{\partial r}+\frac{\partial^{2}\lambda }{\partial r^{2}}\right)\\ \frac{c_{1}}{r}\left(\theta_{r}+\frac{\partial \lambda }{\partial r}\right)\\ 0\\ 0 \end{array}\right\}, \end{array}$$

where $c_{2}=\frac{4}{h^{2}}$. The symmetric part of the curvature tensor for axisymmetric plates is defined as

(13) $$\begin{array}{cc} \chi_{r\theta } & =\frac{1}{2}\left(\frac{\partial \omega_{\theta }}{\partial r}-\frac{\omega_{\theta }}{r}\right)\\ \chi_{\theta z} & =\frac{1}{2}\frac{\partial \omega_{\theta }}{\partial z}, \end{array}$$

where $\omega_{\theta }=\frac{1}{2}\left(\frac{\partial u_{r}}{\partial z}-\frac{\partial u_{z}}{\partial r}\right)$. Thus, the $\chi_{r\theta }$ and $\chi_{\theta z}$ in terms of the displacements in Equation (10) take the form of

(14) $$\begin{array}{cc} \left\{\begin{array}{c} \chi_{r\theta }\\ \chi_{\theta z} \end{array}\right\} & =\frac{1}{4}\left\{\begin{array}{c} \frac{\partial \theta_{r}}{\partial r}-\frac{\partial^{2}w_{0}}{\partial r^{2}}-\frac{1}{r}\left(\theta_{r}-\frac{\partial w_{0}}{\partial r}\right)\\ -2\frac{\partial \theta_{z}}{\partial r} \end{array}\right\}-\frac{z}{2}\left\{\begin{array}{c} \frac{\partial^{2}\theta_{z}}{\partial r^{2}}-\frac{1}{r}\frac{\partial \theta_{z}}{\partial r}\\ c_{2}\left(\theta_{r}+\frac{\partial \lambda }{\partial r}\right)+\frac{\partial \phi_{z}}{\partial r} \end{array}\right\}\\ \; & \;-\frac{z^{2}}{4}\left\{\begin{array}{c} c_{2}\left[\frac{\partial \theta_{r}}{\partial r}+\frac{\partial^{2}\lambda }{\partial r^{2}}-\frac{1}{r}\left(\theta_{r}+\frac{\partial \lambda }{\partial r}\right)\right]+\frac{\partial^{2}\phi_{z}}{\partial r^{2}}-\frac{1}{r}\frac{\partial \phi_{z}}{\partial r}\\ 0. \end{array}\right\}. \end{array}$$

### 3.2. Governing Equations

In this study, the soft-coupled thermoelastical behavior of functionally-graded porous materials was analyzed using the finite element method. The equations of equilibrium and the weak form finite element model for static bending problems of axisymmetric plate were obtained using the principle of virtual displacement.

(15) $$\begin{array}{cc} 0=- & \int_{V}\left(\sigma_{rr}\delta \varepsilon_{rr}+\sigma_{\theta \theta }\delta \varepsilon_{\theta \theta }+\sigma_{zz}\delta \varepsilon_{zz}+2\sigma_{rz}\delta \varepsilon_{rz}+2m_{r\theta }\delta \chi_{r\theta }+2m_{\theta z}\delta \chi_{\theta z}\right)dV\\ \; & \;+\int_{V}\left({\bar{f}}_{i}\delta u_{i}+{\bar{c}}_{i}\delta \omega_{i}\right)dV+\int_{S}\left({\bar{t}}_{i}\delta u_{i}+{\bar{s}}_{i}\delta \omega_{i}\right)dS\\ \; & \;+\int_{\Omega^{t}}\left(q_{i}^{t}\delta u_{i}+p_{i}^{t}\delta \omega_{i}\right)d\Omega^{t}+\int_{\Omega^{b}}\left(q_{i}^{b}\delta u_{i}+p_{i}^{b}\delta \omega_{i}\right)d\Omega^{b}, \end{array}$$

where $\sigma_{ij}$ and $m_{ij}$ are the symmetric part of the stress tensor and the deviatoric part of the couple stress tensor. ${\bar{f}}_{i}$ and ${\bar{c}}_{i}$ are the body forces and couples. ${\bar{t}}_{i}$, ${\bar{s}}_{i}$, and $\bar{d}$ are the surface forces and couples on the side surfaces. $q_{i}^{\alpha }$ and $p_{i}^{\alpha }$ are the surface forces and couples on top $\left(\alpha =t\right)$ and bottom $\left(\alpha =b\right)$ surfaces.

The governing equations of quasi-3D Reddy third order theory are

(16) $$\begin{array}{cc} 0= & \frac{1}{r}\left[\frac{\partial }{\partial r}\left(rN_{rr}^{\left(0\right)}\right)-N_{\theta \theta }^{\left(0\right)}\right]+F_{r}^{\left(0\right)} \end{array}$$

(17) $$\begin{array}{cc} 0= & \frac{1}{r}\left\{\frac{\partial }{\partial r}\left(rN_{rr}^{\left(1\right)}\right)-N_{\theta \theta }^{\left(1\right)}-c_{1}\left(\frac{\partial }{\partial r}\left(rN_{rr}^{\left(3\right)}\right)-N_{\theta \theta }^{\left(3\right)}\right)-r\left(N_{rz}^{\left(0\right)}-c_{2}N_{rz}^{\left(2\right)}\right)\right.\\ \; & \;\left.+\frac{1}{2}\left[\frac{\partial }{\partial r}\left(rM_{r\theta }^{\left(0\right)}\right)+M_{r\theta }^{\left(0\right)}-c_{2}\left(\frac{\partial }{\partial r}\left(rM_{r\theta }^{\left(2\right)}\right)+M_{r\theta }^{\left(2\right)}-2rM_{\theta z}^{\left(1\right)}\right)\right]\right\}\\ \; & \;+F_{r}^{\left(1\right)}-c_{1}F_{r}^{\left(3\right)} \end{array}$$

(18) $$\begin{array}{cc} 0= & \frac{1}{r}\left\{\frac{\partial }{\partial r}\left[rN_{rr}^{\left(0\right)}\left(\frac{\partial w}{\partial r}\right)\right]+c_{1}\frac{\partial }{\partial r}\left[\frac{\partial }{\partial r}\left(rN_{rr}^{\left(3\right)}\right)-rN_{\theta \theta }^{\left(3\right)}\right]+\frac{\partial }{\partial r}\left(rN_{rz}^{\left(0\right)}\right)-c_{2}\frac{\partial }{\partial r}\left(rN_{rz}^{\left(2\right)}\right)\right.\\ \; & \;\left.+\frac{1}{2}\left[\frac{\partial }{\partial r}\left(\frac{\partial }{\partial r}\left(rM_{r\theta }^{\left(0\right)}\right)+M_{r\theta }^{\left(0\right)}\right)+c_{2}\frac{\partial }{\partial r}\left(\frac{\partial }{\partial r}\left(rM_{r\theta }^{\left(2\right)}\right)+M_{r\theta }^{\left(2\right)}\right)-2c_{2}\frac{\partial }{\partial r}\left(rM_{\theta z}^{\left(1\right)}\right)\right]\right\}\\ \; & \;+F_{z}^{\left(0\right)}+\frac{c_{1}}{r}\frac{\partial }{\partial r}\left(rF_{r}^{\left(3\right)}\right) \end{array}$$

(19) $$\begin{array}{cc} 0= & \frac{1}{r}\left\{\frac{1}{2}\frac{\partial }{\partial r}\left[\frac{\partial }{\partial r}\left(rN_{rr}^{\left(2\right)}\right)-N_{\theta \theta }^{\left(2\right)}\right]-N_{zz}^{\left(0\right)}\right.\\ \; & \;\left.+\left[\frac{\partial }{\partial r}\left(\frac{\partial }{\partial r}\left(rM_{r\theta }^{\left(1\right)}\right)+M_{r\theta }^{\left(1\right)}-rM_{\theta z}^{\left(0\right)}\right)\right]\right\}+F_{z}^{\left(1\right)}+\frac{1}{2}\frac{\partial }{\partial r}\left(rF_{r}^{\left(2\right)}\right) \end{array}$$

(20) $$\begin{array}{cc} 0= & \frac{1}{r}\left\{\frac{1}{3}\frac{\partial }{\partial r}\left[\frac{\partial }{\partial r}\left(rN_{rr}^{\left(3\right)}\right)-N_{\theta \theta }^{\left(3\right)}\right]-2rN_{zz}^{\left(1\right)}\right.\\ \; & \;\left.+\frac{\partial }{\partial r}\left[\frac{\partial }{\partial r}\left(rM_{r\theta }^{\left(2\right)}\right)+M_{r\theta }^{\left(2\right)}-2rM_{\theta z}^{\left(1\right)}\right]\right\}+F_{z}^{\left(2\right)}+\frac{1}{3}\frac{\partial }{\partial r}\left(rF_{r}^{\left(3\right)}\right), \end{array}$$

where $\left\{N_{ij}^{\left(k\right)},M_{ij}^{\left(k\right)}\right\}=\int_{-\frac{h}{2}}^{\frac{h}{2}}z^{k}\left\{\sigma_{ij},m_{ij}\right\}dz$ and $F_{i}^{\left(k\right)}=\int_{-\frac{h}{2}}^{\frac{h}{2}}z^{k}\left\{f_{i}+\left[q_{i}^{t}-1^{k}q_{i}^{b}\right]\right\}dz$. Note that the body couple ${\bar{c}}_{\theta }$ is omitted in the governing equation.

The temperature distribution through thickness direction can be determined by solving the steady state energy equation,

(21) $$-\frac{d}{dz}\left(k\left(z\right)\frac{dT}{dz}\right)=0,$$

where k(z) is heat conductivity and *T* is the temperature. The effective thermal conductivity is defined using the Maxwell–Eucken model described by Deng et al. [51]:

(22) $$k\left(z\right)=k_{s}\left(z\right)\left[\frac{k_{f}+2k_{s}\left(z\right)+2\Phi \left(k_{f}-k_{s}\left(z\right)\right)}{k_{f}+2k_{s}\left(z\right)-\Phi \left(k_{f}-k_{s}\left(z\right)\right)}\right],$$

where $k_{s}\left(z\right)$ and $k_{f}$ are the thermal conductivity of the solid and fluid phases, respectively, and Φ is the porosity. In this study, the thermal conductivity of the solid is obtained using a power-law distribution described in previous section.

## 4. Finite Element Model

A weak from Galerkin finite element model for the circular plate bending is developed using the principle of virtual displacement (15) and a weak form is directly developed from the energy Equation (21) for steady state heat conduction problem. The details of weak form Galerkin finite element model can be found in Reddy [52].

The temperature *T* and generalized displacements $\left(u_{0},\theta_{r},w_{0},\theta_{z},\phi_{z}\right)$ are approximated in following form:

(23) $$\begin{array}{cc} T\left(z\right)= & \sum_{j=1}^{n}T_{j}{\hat{\psi }}_{j}\left(z\right) \end{array}$$

(24) $$\begin{array}{cc} u_{0}\left(r\right)= & \sum_{j=1}^{n}u_{j}\psi_{j}\left(r\right) \end{array}$$

(25) $$\begin{array}{cc} \theta_{r}\left(r\right)= & \sum_{j=1}^{n}\theta_{j}\psi_{j}\left(r\right) \end{array}$$

(26) $$\begin{array}{cc} w_{0}\left(r\right)= & \sum_{J=1}^{2n}\Delta_{J}^{\left(1\right)}\phi_{J}\left(r\right) \end{array}$$

(27) $$\begin{array}{cc} \theta_{z}\left(r\right)= & \sum_{J=1}^{2n}\Delta_{J}^{\left(2\right)}\phi_{J}\left(r\right) \end{array}$$

(28) $$\begin{array}{cc} \phi_{z}\left(r\right)= & \sum_{J=1}^{2n}\Delta_{J}^{\left(3\right)}\phi_{J}\left(r\right), \end{array}$$

where $T_{j}$ are nodal temperatures through thickness direction; $u_{j},\theta_{j},$ and $w_{j}$ are nodal displacements in the radial direction; ${\hat{\psi }}_{j}$ and $\psi_{j}$ are the Lagrange interpolation functions; $\phi_{J}$ are the Hermite interpolation functions; $\Delta_{J}^{\left(i\right)}$ are generalized deflections and i=1,2,3 correspond to $w_{0},\theta_{z},\phi_{z}$, respectively; *n* is the number of nodes in an element.

The finite element model of the steady state heat conduction problem is given by

(29) $${\left[\begin{array}{c} \hat{K} \end{array}\right]}^{e}\left\{\begin{array}{c} T^{e} \end{array}\right\}={\left\{\begin{array}{c} \hat{F} \end{array}\right\}}^{e},$$

where the stiffness matrix and external heat flux are

(30) $$\begin{array}{cc} {\hat{K}}_{ij} & =\int_{-\frac{h}{2}}^{\frac{h}{2}}k\left(z\right)\left(\frac{d{\hat{\psi }}_{i}}{dz}\frac{d{\hat{\psi }}_{i}}{dz}\right)dz \end{array}$$

(31) $$\begin{array}{cc} {\hat{F}}_{i} & =q_{i}. \end{array}$$

The finite element model of an axisymmetric plate static bending is given by

(32) $${\left[\begin{array}{ccccc} K^{11} & K^{12} & K^{13} & K^{14} & K^{15}\\ K^{21} & K^{22} & K^{23} & K^{24} & K^{25}\\ K^{31} & K^{32} & K^{33} & K^{34} & K^{35}\\ K^{41} & K^{42} & K^{43} & K^{44} & K^{45}\\ K^{51} & K^{52} & K^{53} & K^{54} & K^{55} \end{array}\right]}^{e}{\left\{\begin{array}{c} \left\{u_{0}\right\}\\ \left\{\theta_{r}\right\}\\ \left\{w_{0}\right\}\\ \left\{\theta_{z}\right\}\\ \left\{\phi_{z}\right\} \end{array}\right\}}^{e}={\left\{\begin{array}{c} \left\{F^{1}\right\}\\ \left\{F^{2}\right\}\\ \left\{F^{3}\right\}\\ \left\{F^{4}\right\}\\ \left\{F^{5}\right\} \end{array}\right\}}^{e}.$$

The elements of the stiffness matrix, $K^{lm}$, and the elements of force vector, $F^{l}$, are defined in Appendix A.

The solution of the nonlinear finite element model (32) is obtained using Newton’s iteration procedure. The linearized element equations take the form of

(33) $$T^{e}\left(\Delta^{\left(i-1\right)}\right)\delta \Delta^{\left(i\right)}=-R^{e}\left(\Delta^{\left(i-1\right)}\right),$$

where $T^{e}$ is the tangent stiffness matrix, $\delta \Delta^{\left(i\right)}$ is incremental displacements at the *i*th iteration, and $R^{e}$ is the residual vector. The tangent matrix and residual are defined as [52]

(34) $$T^{e}=\frac{\partial R^{e}}{\partial \Delta^{e}},\;R^{e}={\left(K^{e}\Delta^{e}-F^{e}\right)}^{\left(i-1\right)}.$$

By solving the assembled global system equation, the global incremental displacement vector at *i*th iteration, $\delta U^{\left(i\right)}$ is obtained.

(35) $$\delta U^{\left(i\right)}=-{\left[T\left(U^{\left(i-1\right)}\right)\right]}^{-1}R^{\left(i-1\right)}.$$

The total displacement at the *i*th iteration is obtained by adding the incremental solution at the *i*th iteration to the previous solution at the (i−1)th iteration [39].

(36) $$U^{\left(i\right)}=U^{\left(i-1\right)}+\delta U^{\left(i\right)}.$$

In this study, we considered geometrical nonlinearity with elastic material behavior. For this purpose, the Newton’s iteration is sufficient to obtain the converged solutions. However, when limit load, softening branches, or snap-through behavior are considered, another solution procedure, such as the arc length method, should be considered. These solution procedures can be used in conjunction with various numerical methods such as isogeometric techniques [53,54] or the Rayleigh Ritz method [55] in addition to the finite element method.

## 5. Numerical Results

In the numerical examples, we considered several examples of annular circular plates with various boundary conditions such as simply-supported and clamped boundary conditions. To validate the developed finite element model, we compared our results with available studies in the literature. We also conducted convergence studies to obtain optimal mesh size and different quadrature rules to make sure we avoided any locking phenomena. In this study, we used 16 elements and full quadrature rules for linear parts of the stiffness matrix and reduced quadrature rules for shear, nonlinear, and couple stress parts of the stiffness matrix.

**Figure 2:**
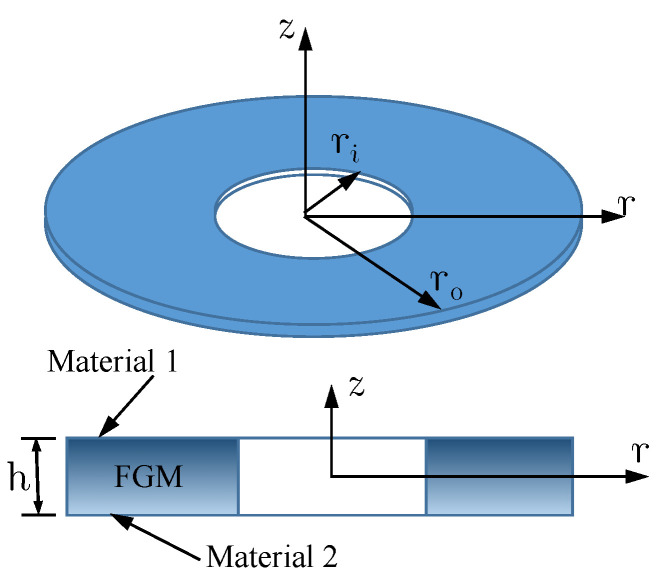
An axisymmetric FGM annular plate [39].

Figure 2 shows the annular plate we studied. The numerical parameters for the validation study were adapted from the study of Reddy et al. [39]: *h* = 0.1, $r_{o}=$ 10*h*, $r_{i}=0.25r_{0}$, $E_{1}=10^{6}$, and $E_{2}=10^{5}$.

**Figure 3:**
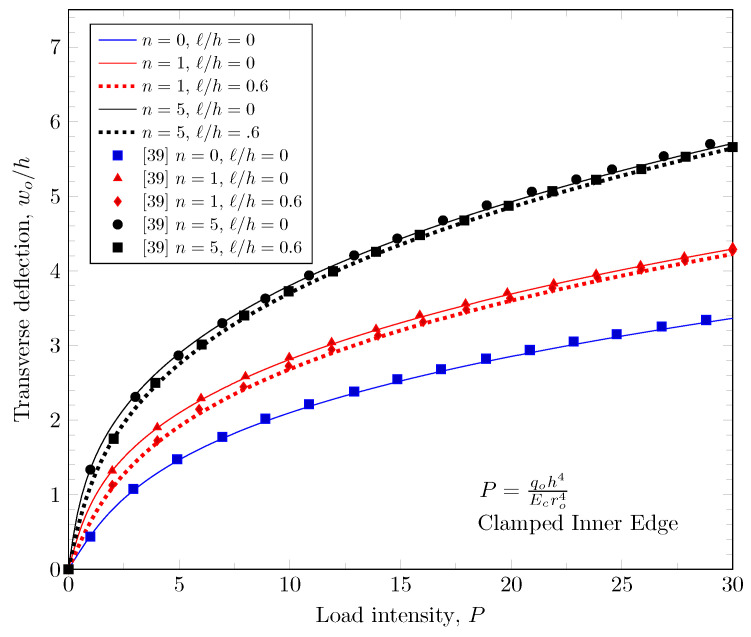
Maximum deflection at outer edge with clamped inner edge.

**Figure 4:**
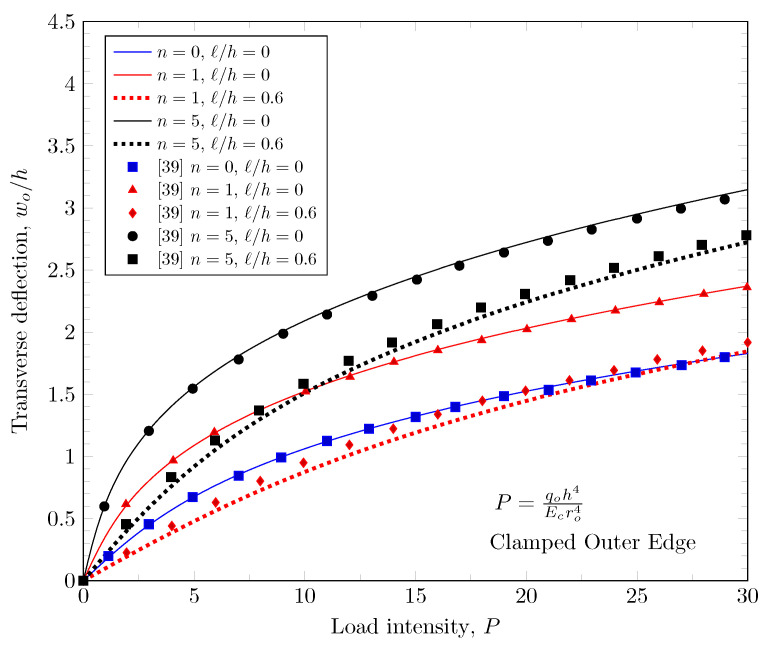
Maximum deflection at inner edge with clamped outer edge.

**Figure 5:**
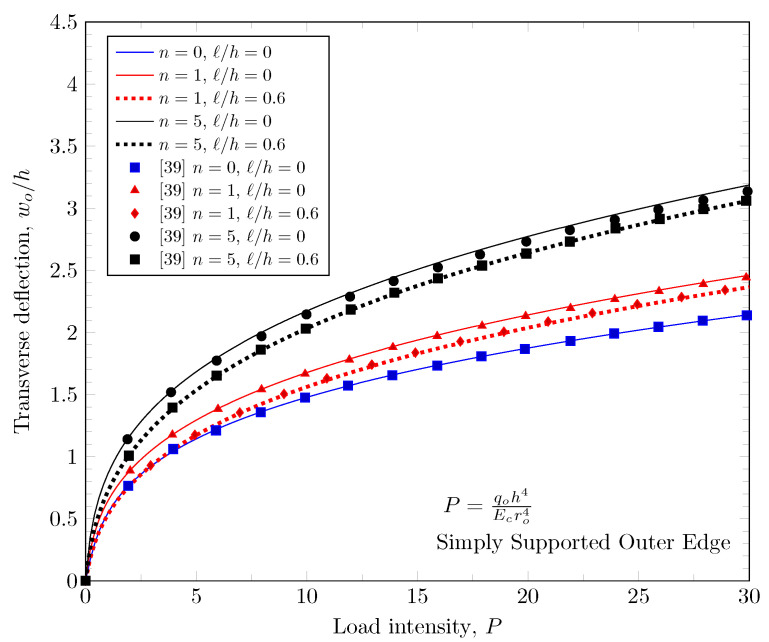
Maximum deflection at inner edge with simply-supported outer edge.

Figure 3, Figure 4 and Figure 5 show that maximum deflection versus the load parameter $P=\frac{q_{0}h^{4}}{E_{c}r_{o}^{4}}$ at the free edges, where $q_{0}$ is a distributed load on the top surface, *h* is the plate thickness, $E_{c}$ is the Young’s modulus of ceramic materials on the top surface, and $r_{o}$ is the outer radius of the annular plate. The developed finite element model shows a good agreement with the study of Reddy et al. [39]. In this figure, the effects of the material variations based on the change of the power-law index, and the effect of length scale parameter are presented with various boundary conditions.

With the validated finite element model, we evaluated the effects of various parameters such as the length scale parameters, the shape of porosity distribution, power law index, and boundary conditions. In this study, we considered a porous functionally-graded material with Monel and zirconia and the material properties of them follow [56]:

$$\begin{array}{cc} K_{m} & =227.24\;\mathrm{GPa},\;\mu_{m}=65.55\;\mathrm{GPa},\;\alpha_{m}=15\times 10^{-6}\;/K,\;k_{m}=25\;W/\mathrm{mK}\;\mathrm{for}\;\mathrm{Monel}\\ K_{c} & =125.83\;\mathrm{GPa},\;\mu_{c}=58.077\;\mathrm{GPa},\;\alpha_{c}=10\times 10^{-6}\;/K,\;k_{c}=2.09\;W/\mathrm{mK}\;\mathrm{for}\;\mathrm{zirconia}, \end{array}$$

where $K_{i}$ is the bulk modulus, $\mu_{i}$ is the shear modulus, $\alpha_{i}$ is the thermal expansion coefficient, $k_{i}$ is the thermal conductivity, and the subscription *m* and *c* indicate metal and ceramic, respectively. We assumed that the porous is filled with the air and the thermal conductivity of the air is assumed to be $k_{a}=0.025572\;W/\mathrm{mK}$.

**Figure 6:**
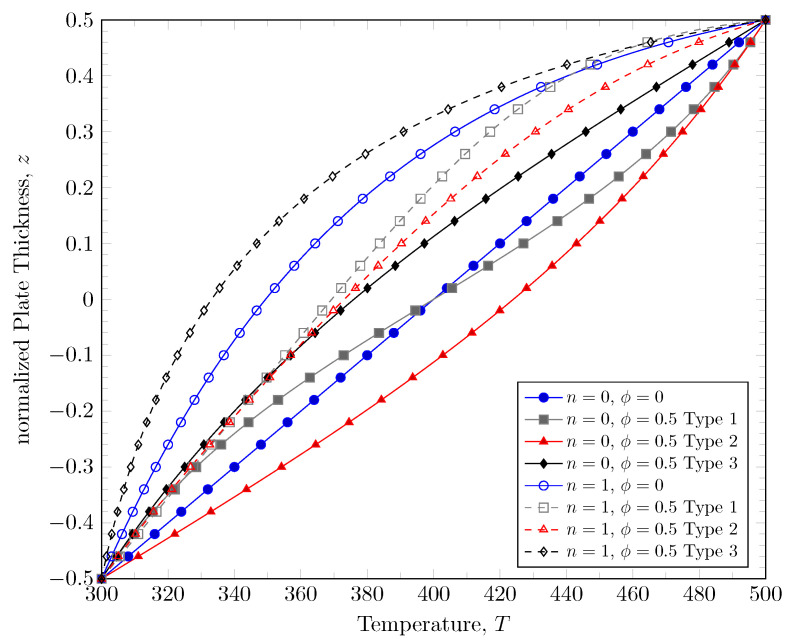
Temperature distribution of porous FGM.

To induce thermal load, two different temperatures were applied on the top and bottom surfaces; 500 K was applied on the top surface and 300 K was applied on the bottom surface. Figure 6 shows the temperature distribution through plate thickness depending on the variation of material constituents and porosity distribution. The temperature distribution was obtained by solving the energy Equation (21). Three different types of porosity distributions and the variation of material constituents were considered. In the area where the volume fraction for porosity is larger, the thermal resistance becomes larger and the temperature change through the thickness is less than the area where the volume fraction of porosity is lesser. With a larger power-law index, the effective thermal conductivity is increased and thermal resistance becomes smaller because the volume fraction of metal is increased.

For illustration purposes, the same plate geometry as Reddy et al. [39] was used, and the effects of various parameters with clamped and simply-supported boundary conditions were considered.

**Figure 7:**
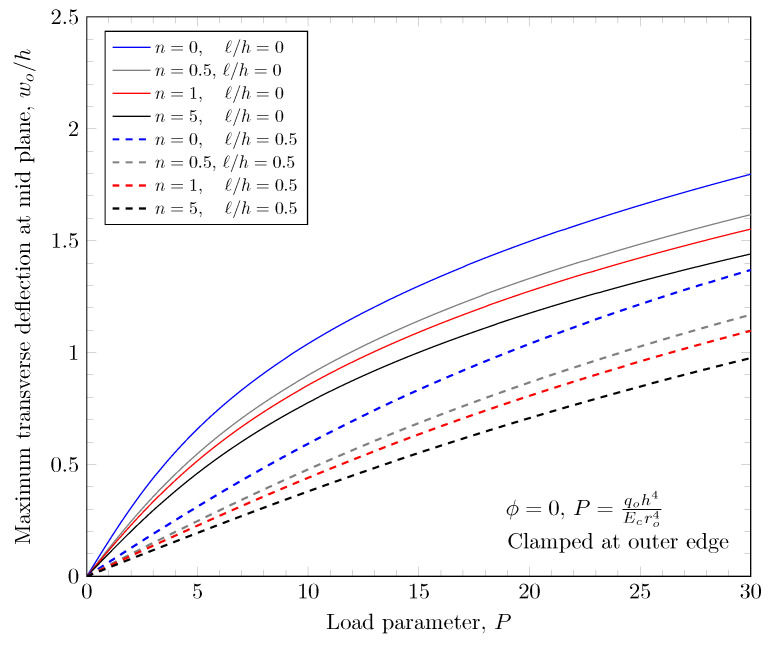
Maximum deflection of FGM with clamped outer edge with nonzero length scale parameters.

**Figure 8:**
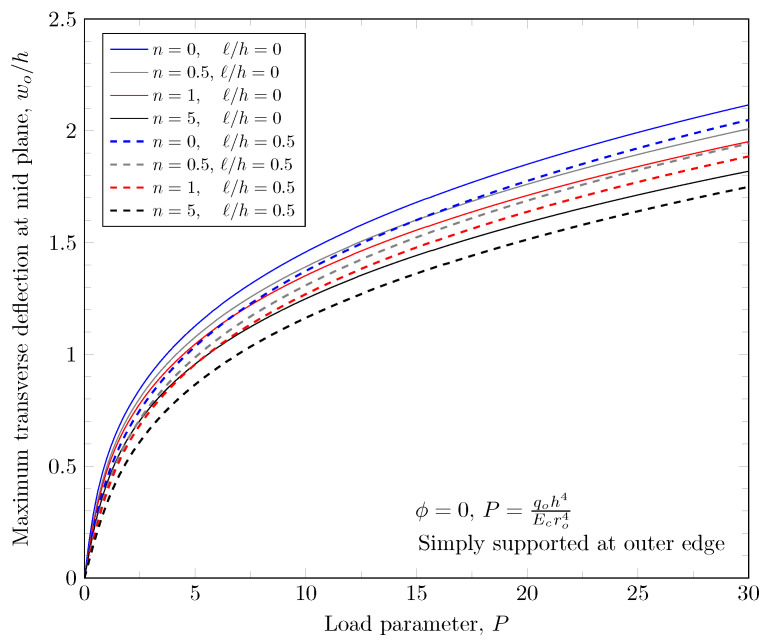
Maximum deflection of FGM with simply-supported outer edge with nonzero length scale parameters.

**Figure 9:**
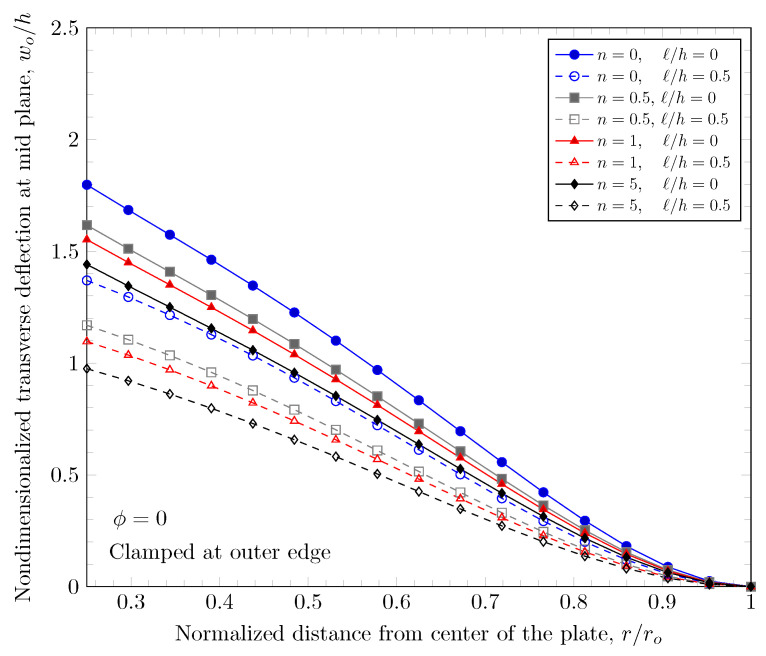
Deflection of FGM with clamped outer edge with nonzero length scale parameters.

**Figure 10:**
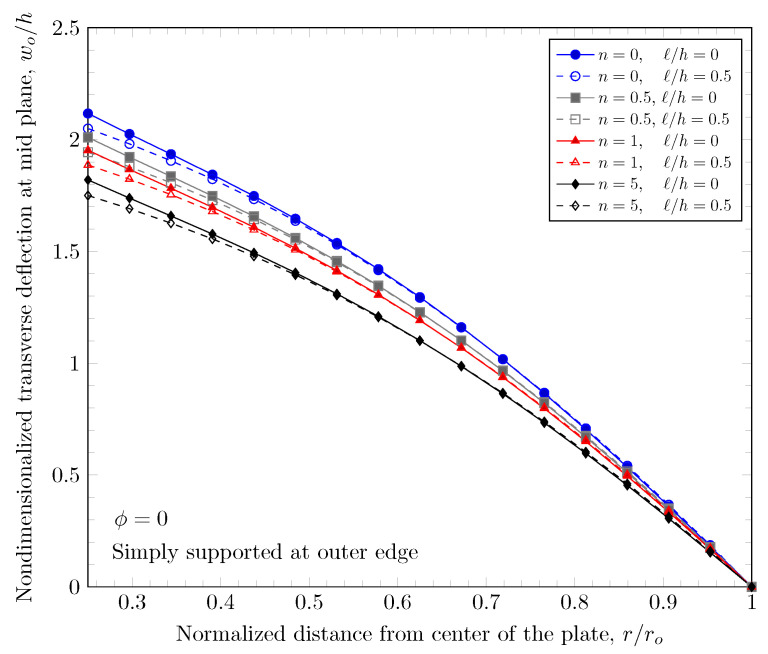
Deflection of FGM with simply-supported outer edge with nonzero length scale parameters.

Figure 7 and Figure 8 show effects of the length scale parameter on the maximum deflection of FGM plates. When the power-law index is larger, the volume fraction of Monel is larger and the FGM plate becomes stiffer. The length scale parameter can capture the stiffening behavior in micro scale structures. The deflections of FGM plates with various length scale parameters are shown in Figure 9 and Figure 10.

**Figure 11:**
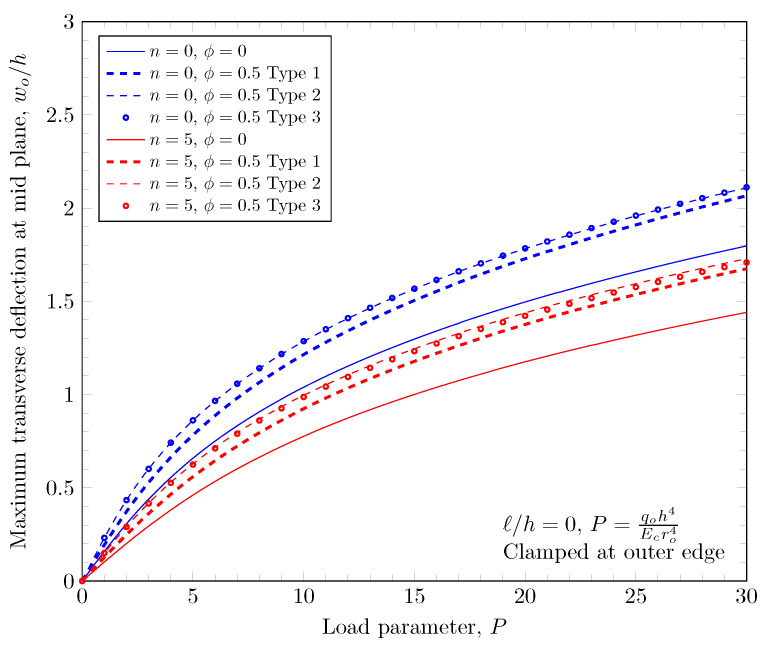
Maximum deflection of FGM with clamped outer edge with nonzero porosity.

**Figure 12:**
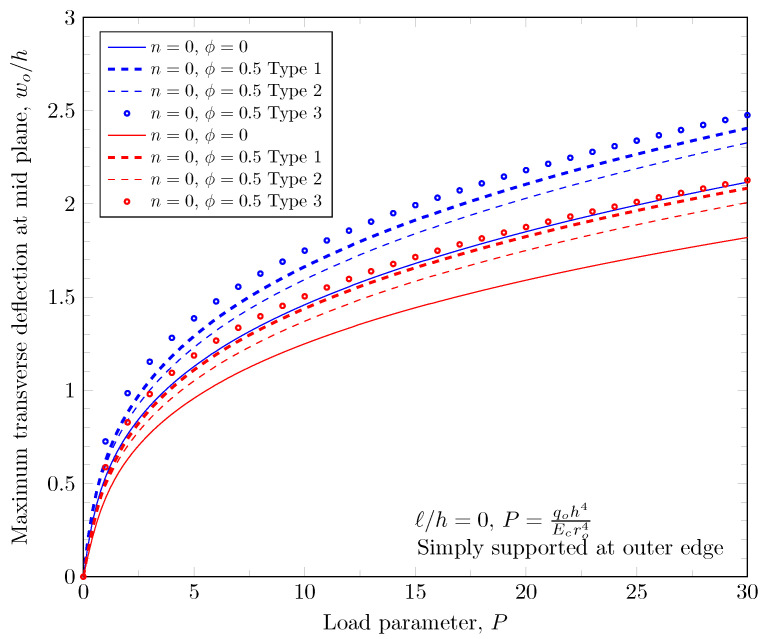
Maximum deflection of FGM with simply-supported outer edge with nonzero porosity.

**Figure 13:**
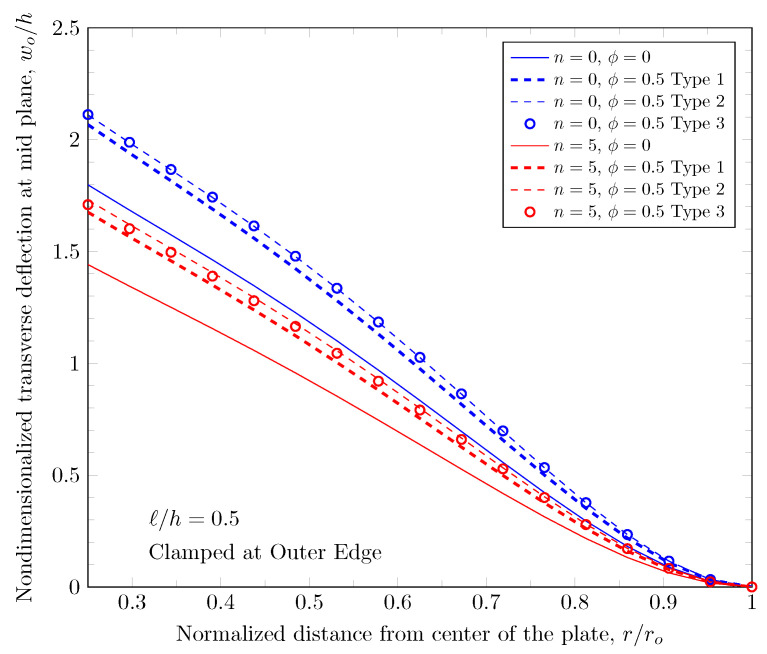
Deflection of FGM with clamped outer edge with nonzero porosity.

**Figure 14:**
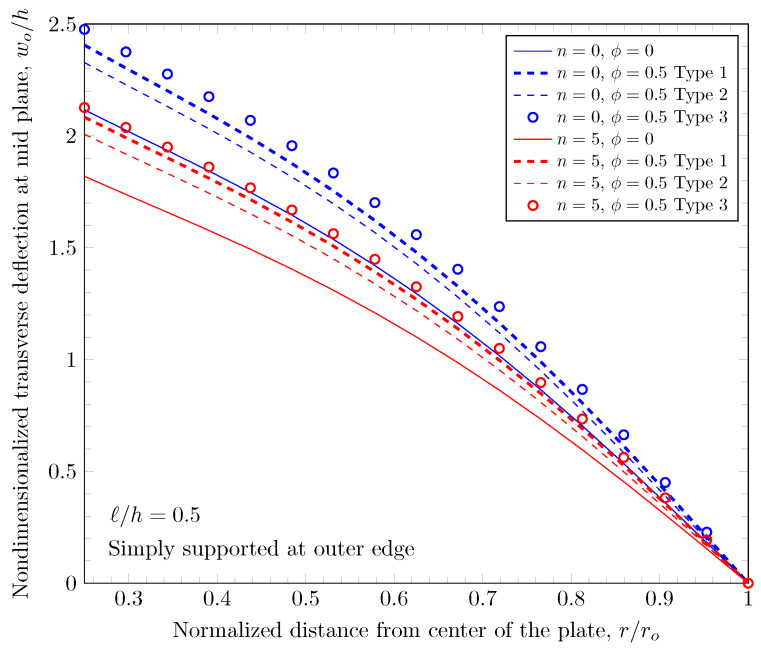
Deflection of FGM with simply-supported outer edge with nonzero porosity.

The overall volume fraction of porosity in three porosity distributions is the same, but the enhanced porous areas are mid, bottom, and top surfaces with Type 1, Type 2, and Type 2 porosity distributions. The porous FGM is softer than non-porous FGM, and Type 1 results in the stiffest plates because the materials on the top and bottom surfaces remain. There are no differences in the plate bending stiffness between Type 1 and Type 2 distribution when a homogeneous material is assumed. When the power-law index is larger than zero, the volume fraction of stiffer material becomes larger in the FGMs. In the case of Type 2 distribution, the volume fraction of the stiffer material is decreased, and in the case of Type 3, the volume fraction of the softer material is decreased. Therefore, Type 2 will be softer than Type 3 in the case of FGMs. Figure 11 and Figure 12 show the effects of three different porosity distributions and material variations on the maximum deflections with clamped and simply-supported outer edges, respectively. The deflections along the radial direction are shown in Figure 13 and Figure 14.

**Figure 15:**
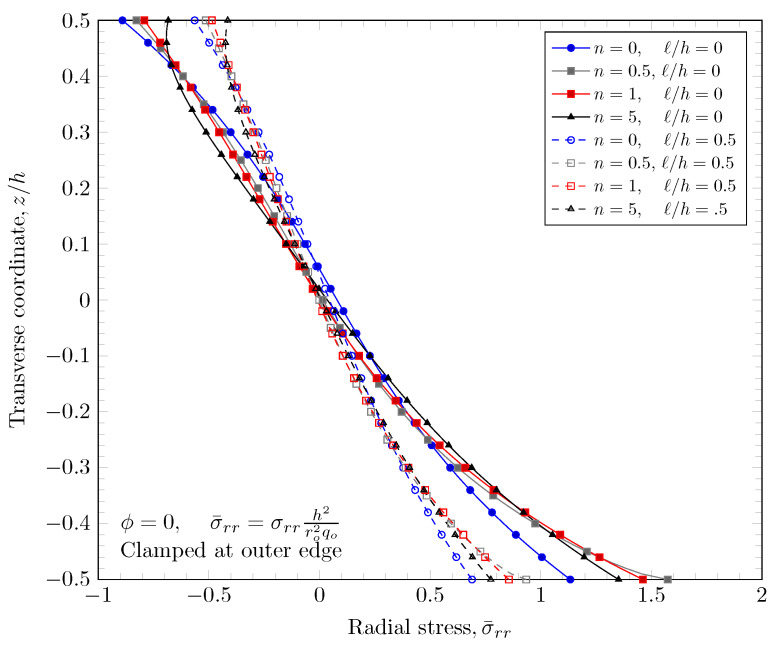
Normal stress variation through the thickness of plate with clamped outer edge with nonzero length scale parameters.

**Figure 16:**
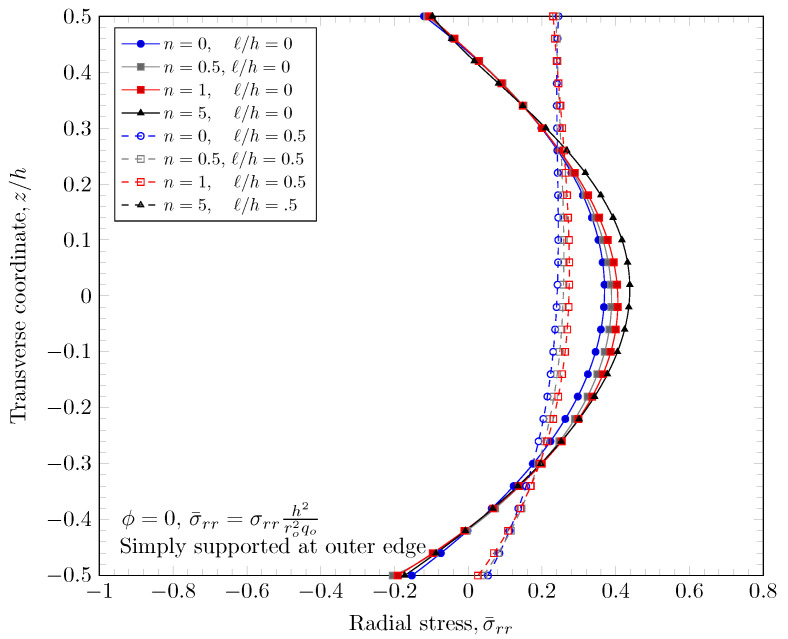
Normal stress variation through the thickness of plate with simply-supported outer edge with nonzero length scale parameters.

**Figure 17:**
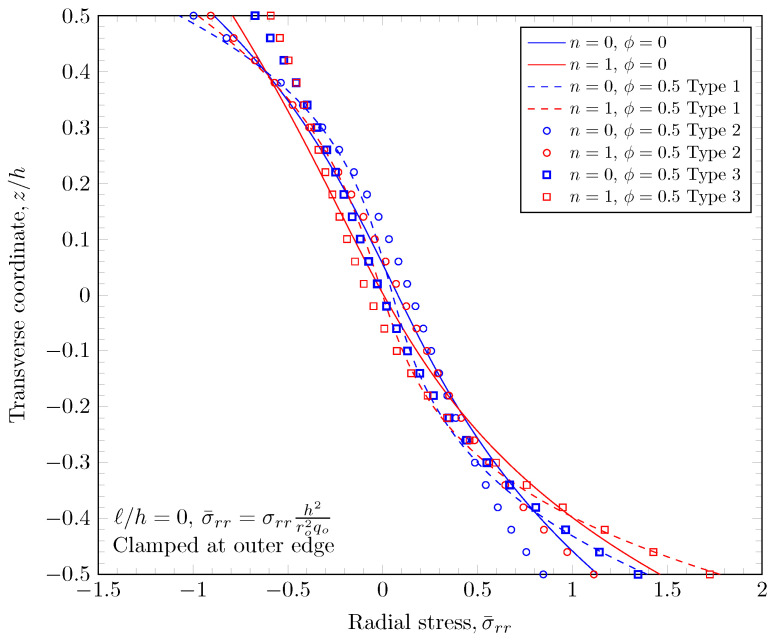
Normal stress variation through the thickness of plate with clamped outer edge with nonzero porosity.

**Figure 18:**
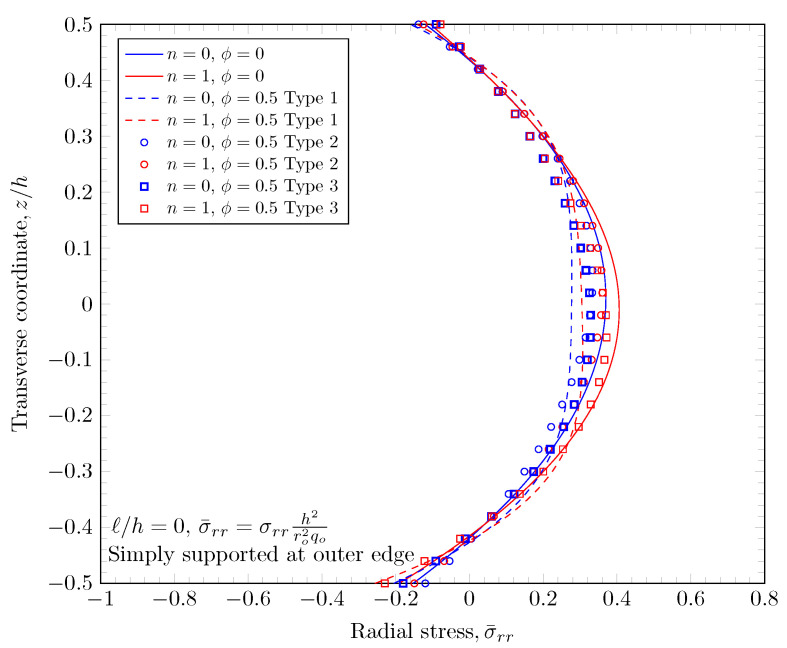
Normal stress variation through the thickness of plate with simply-supported outer edge porosity.

Figure 15, Figure 16, Figure 17 and Figure 18 show a normal stress distribution through the plate thickness. In the case of the clamped outer edge, the normal stress in the area where the volume fraction of porosity is larger is smaller than the area where the volume fraction of porosity is smaller because the area with larger porosity is softer than the other areas. It is clearly shown that the normal stress at the bottom surface (z=−h/2) with porosity distribution Type 3 is larger than porosity distribution Type 2, which enhances the porosity distribution in the lower area of the plates. In the case of the simply-supported outer edge, the normal stress distribution is a parabolic shape unlike the case of the clamped outer edge. This is because the thickness stretch is not constrained in the case of simply-supported boundary conditions. Only the mid plane deflection, $w_{0}$, is constrained. The nonzero length scale parameters make the FGM plate stiffer, but there are no material property changes, which results in smaller stresses.

**Figure 19:**
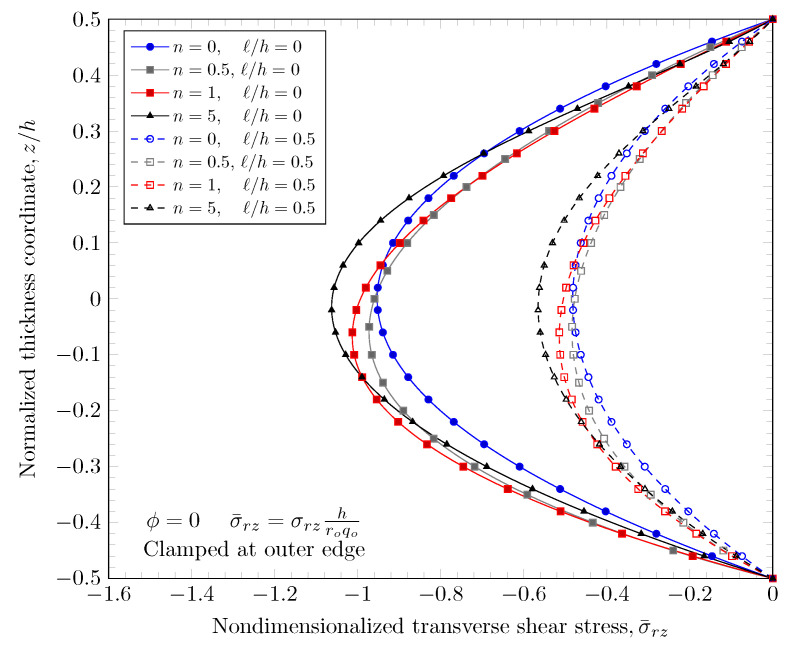
Transverse stress variation through the thickness of plate with clamped outer edge with nonzero length scale parameters.

**Figure 20:**
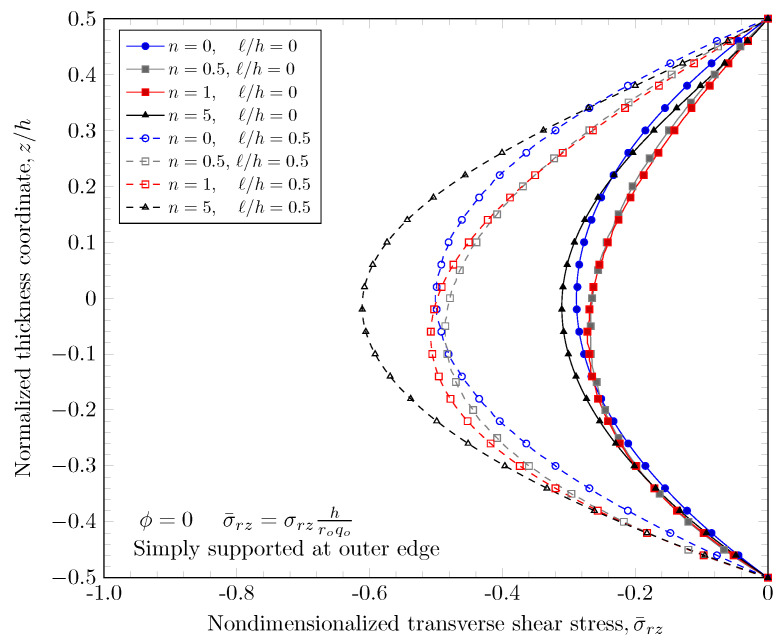
Transverse stress variation through the thickness of plate with simply-supported outer edge with nonzero length scale parameters.

**Figure 21:**
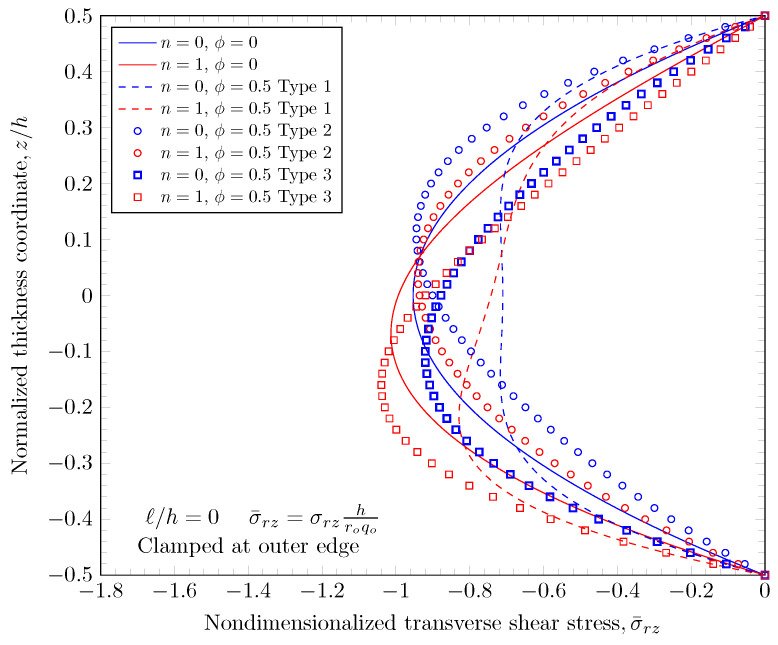
Transverse stress variation through the thickness of plate with clamped outer edge with nonzero porosity.

**Figure 22:**
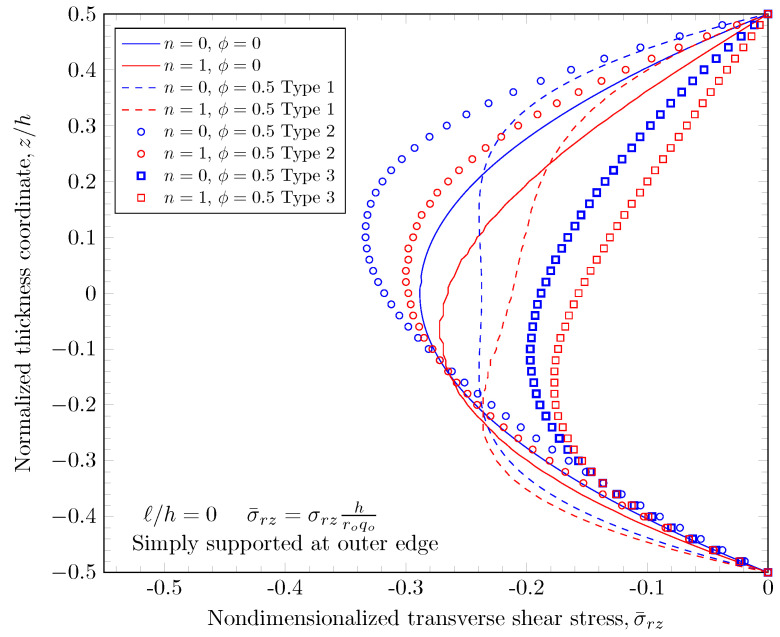
Transverse stress variation through the thickness of plate with simply-supported outer edge porosity.

Figure 19, Figure 20, Figure 21 and Figure 22 show transverse shear stress distributions through the plate thickness. Similar effects of porosity distribution and length scale parameter on the transverse shear stresses are observed. The length scale parameter makes the FGM plate stiffer and smaller stress values are obtained. However, the porosity distribution affects the material properties and larger stress values are present in stiffer areas. The proposed quasi-3D Reddy third-order plate theory can capture a parabolic variation of the transverse shear stresses, and it does not require a shear correction factor that is present in low order shear deformation theories.

**Figure 23:**
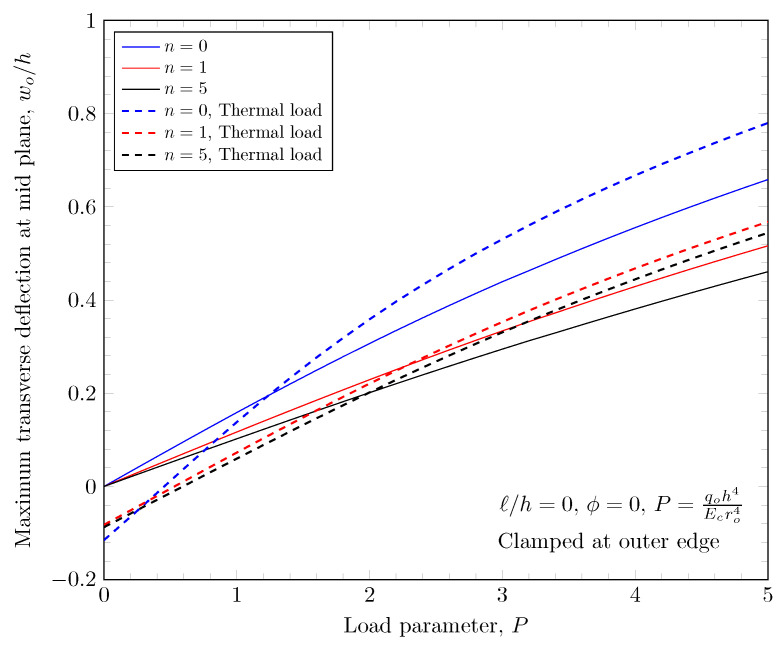
Maximum deflection of FGM under thermal load with clamped outer edge.

**Figure 24:**
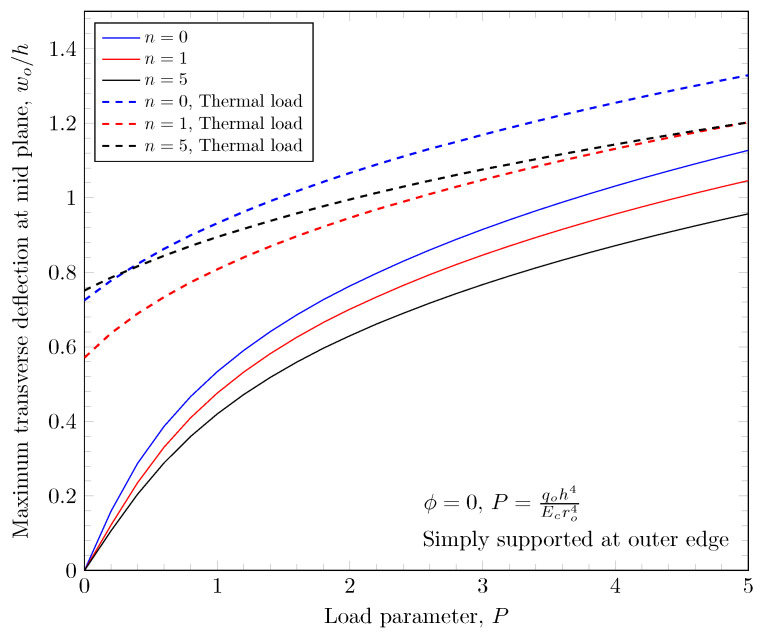
Maximum deflection of FGM under thermal load with simply-supported outer edge.

**Figure 25:**
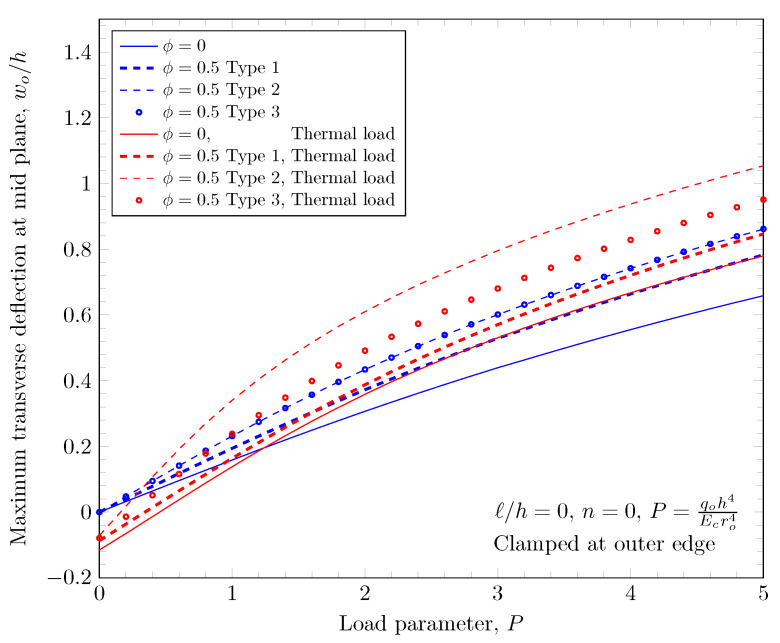
Maximum deflection of porous FGM under thermal load with clamped outer edge.

**Figure 26:**
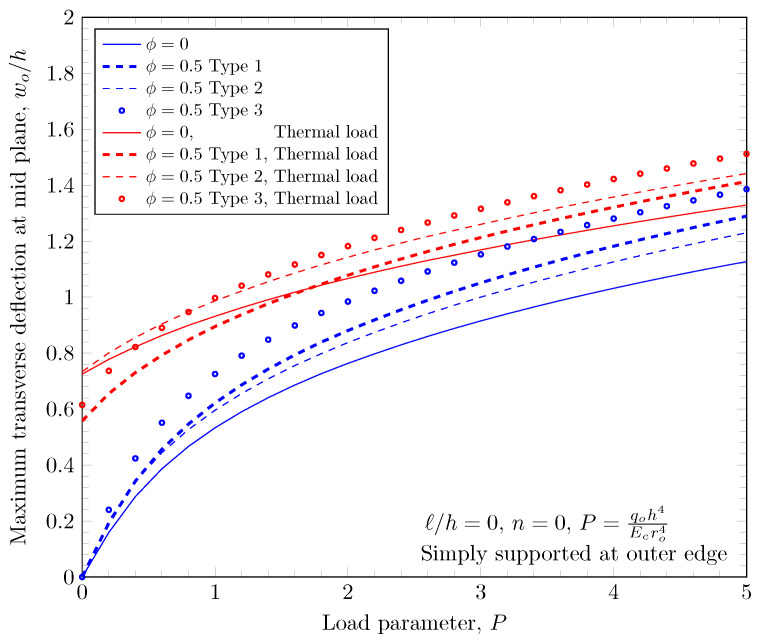
Maximum deflection of porous FGM under thermal load with simply-supported outer edge.

Figure 23, Figure 24, Figure 25 and Figure 26 show the effect of thermal load. The thermal load is induced by temperature boundary conditions; 500 K is applied on top surface and 300 K is applied on bottom surfaces. In the case of the clamped outer edge, the deflection is due to thermal load in the negative direction because the plate bends down due to the thermal load. This is clearly shown in Figure 25. In the case of the simply-supported outer edge, the plate bends down due to the thermal load at the same place; the plate rotates about the outer edge which results in the positive deflection due to the thermal load.

## 6. Conclusions

In this study, a nonlinear finite element (FE) model for axisymmetric circular/annular plates was presented. The developed finite element model accounts for geometric nonlinearity, variation of material constituents, microstructure size effects, and effects of porosity distributions. Using the developed FE model, the bending behavior of functionally-graded axisymmetric annular plates under thermomechanical loads was analyzed.

Numerical analysis results for an axisymmetric annular plate with various boundary conditions were presented. A parametric study was conducted to understand the effects of porosity distributions, the variation of material properties, and microstructure size on the bending behavior of axisymmetric annular plates. In summary, the following results were observed:

- The presence of pores results in higher thermal resistance and reduces the temperature variation;
- With a larger power law index, the plate becomes stiffer because the stiffer material is placed on the bottom surface;
- The length scale parameter can capture stiffening effects in microstructures. The stiffening effect does not change the material properties, so stress values are decreased with nonzero length scale parameters;
- The thermal and mechanical behavior of FGM plates highly depends on the porosity distribution type. The presence of pores makes the plate softer by reducing the moduli, resulting in smaller stress values;
- Depending on the boundary conditions, thermal loads can result in opposite deflections due to constrained rotational degrees of freedom.

The presented finite element model can be extended to an asymmetric circular/annular plate.

## Data Availability

The data presented in this study are available on request from the corresponding author.

## Appendix A

(A1) $$\begin{array}{cc} K_{ij}^{11} & =\int_{r_{1}}^{r_{2}}\left[A_{11}^{\left(0\right)}\left(\frac{\partial \psi_{i}}{\partial r}\frac{\partial \psi_{j}}{\partial r}+\frac{1}{r^{2}}\psi_{i}\psi_{j}\right)+\frac{A_{12}^{\left(0\right)}}{r}\left(\frac{\partial \psi_{i}}{\partial r}\psi_{j}+\psi_{i}\frac{\partial \psi_{j}}{\partial r}\right)\right]rdr \end{array}$$

(A2) $$\begin{array}{cc} K_{ij}^{12} & =\int_{r_{1}}^{r_{2}}\left[\left(A_{11}^{\left(1\right)}-c_{1}A_{11}^{\left(3\right)}\right)\left(\frac{\partial \psi_{i}}{\partial r}\frac{\partial \psi_{j}}{\partial r}+\frac{1}{r^{2}}\psi_{i}\psi_{j}\right)\right.\\ \; & \;\left.+\frac{1}{r}\left(A_{12}^{\left(1\right)}-c_{1}A_{12}^{\left(3\right)}\right)\left(\frac{\partial \psi_{i}}{\partial r}\psi_{j}+\psi_{i}\frac{\partial \psi_{j}}{\partial r}\right)\right]rdr \end{array}$$

(A3) $$\begin{array}{cc} K_{iJ}^{13} & =\int_{r_{1}}^{r_{2}}\left[\frac{A_{11}^{\left(0\right)}}{2}\left(\frac{\partial w_{0}}{\partial r}\right)\frac{\partial \psi_{i}}{\partial r}\frac{\partial \phi_{J}}{\partial r}-c_{1}A_{11}^{\left(3\right)}\left(\frac{\partial \psi_{i}}{\partial r}\frac{\partial^{2}\phi_{J}}{\partial r^{2}}+\frac{1}{r^{2}}\psi_{i}\frac{\partial \phi_{J}}{\partial r}\right)\right.\\ \; & \;\left.+\frac{A_{12}^{\left(0\right)}}{2r}\left(\frac{\partial w_{0}}{\partial r}\right)\psi_{i}\frac{\partial \phi_{J}}{\partial r}-c_{1}\frac{A_{12}^{\left(3\right)}}{r}\left(\frac{\partial \psi_{i}}{\partial r}\frac{\partial \phi_{J}}{\partial r}+\psi_{i}\frac{\partial^{2}\phi_{J}}{\partial r^{2}}\right)\right]rdr \end{array}$$

(A4) $$\begin{array}{cc} K_{iJ}^{14} & =\int_{r_{1}}^{r_{2}}\left[-\frac{A_{11}^{\left(2\right)}}{2}\left(\frac{\partial \psi_{i}}{\partial r}\frac{\partial^{2}\phi_{J}}{\partial r^{2}}+\frac{1}{r^{2}}\psi_{i}\frac{\partial \phi_{J}}{\partial r}\right)+A_{12}^{\left(0\right)}\left(\frac{\partial \psi_{i}}{\partial r}\phi_{J}+\frac{1}{r}\psi_{i}\phi_{J}\right)\right.\\ \; & \;\left.-\frac{A_{12}^{\left(2\right)}}{2r}\left(\frac{\partial \psi_{i}}{\partial r}\frac{\partial \phi_{J}}{\partial r}+\psi_{i}\frac{\partial^{2}\phi_{J}}{\partial r^{2}}\right)\right]rdr \end{array}$$

(A5) $$\begin{array}{cc} K_{iJ}^{15} & =\int_{r_{1}}^{r_{2}}\left[-\frac{A_{11}^{\left(3\right)}}{3}\left(\frac{\partial \psi_{i}}{\partial r}\frac{\partial^{2}\phi_{J}}{\partial r^{2}}+\frac{1}{r^{2}}\psi_{i}\frac{\partial \phi_{J}}{\partial r}\right)+2A_{12}^{\left(1\right)}c_{2}\left(\frac{\partial \psi_{i}}{\partial r}\phi_{J}+\frac{1}{r}\psi_{i}\phi_{J}\right)\right.\\ \; & \;\left.-\frac{A_{12}^{\left(3\right)}}{3r}\left(\frac{\partial \psi_{i}}{\partial r}\frac{\partial \phi_{J}}{\partial r}+\psi_{i}\frac{\partial^{2}\phi_{J}}{\partial r^{2}}\right)\right]rdr \end{array}$$

(A6) $$\begin{array}{cc} K_{ij}^{21} & =\left[\left(A_{11}^{\left(1\right)}-c_{1}A_{11}^{\left(3\right)}\right)\left(\frac{\partial \psi_{i}}{\partial r}\frac{\partial \psi_{j}}{\partial r}+\frac{1}{r^{2}}\psi_{i}\psi_{j}\right)\right.\\ \; & \;\left.+\frac{1}{r}\left(A_{12}^{\left(1\right)}-c_{1}A_{12}^{\left(3\right)}\right)\left(\frac{\partial \psi_{i}}{\partial r}\psi_{j}+\psi_{i}\frac{\partial \psi_{j}}{\partial r}\right)\right]rdr \end{array}$$

(A7) $$\begin{array}{cc} K_{ij}^{22} & =\int_{r_{1}}^{r_{2}}\left\{\left(A_{11}^{\left(2\right)}-2c_{1}A_{11}^{\left(4\right)}+c_{1}^{2}A_{11}^{\left(6\right)}\right)\left(\frac{\partial \psi_{i}}{\partial r}\frac{\partial \psi_{j}}{\partial r}+\frac{1}{r^{2}}\psi_{i}\psi_{j}\right)\right.\\ \; & \;+\frac{1}{r}\left(A_{12}^{\left(2\right)}-2c_{1}A_{12}^{\left(4\right)}+c_{1}^{2}A_{12}^{\left(6\right)}\right)\left(\frac{\partial \psi_{i}}{\partial r}\psi_{j}+\psi_{i}\frac{\partial \psi_{j}}{\partial r}\right)\\ \; & \;+\left(A_{55}^{\left(0\right)}-2c_{2}A_{55}^{\left(2\right)}+c_{2}^{2}A_{55}^{\left(4\right)}\right)\psi_{i}\psi_{j}\\ \; & \;+\frac{1}{4}\left[\frac{1}{r^{2}}\left(B^{\left(0\right)}-2c_{2}B^{\left(2\right)}+c_{2}^{2}B^{\left(4\right)}\right)\left(r^{2}\frac{\partial \psi_{i}}{\partial r}\frac{\partial \psi_{j}}{\partial r}+\psi_{i}\psi_{j}\right)+4c_{2}^{2}B^{\left(2\right)}\psi_{i}\psi_{j}\right.\\ \; & \;\left.\left.-\frac{1}{r}\left(B^{\left(0\right)}-2c_{2}B^{\left(2\right)}+c_{2}^{2}B^{\left(4\right)}\right)\left(\psi_{i}\frac{\partial \psi_{j}}{\partial r}+\frac{\partial \psi_{i}}{\partial r}\psi_{j}\right)\right]\right\}rdr \end{array}$$

(A8) $$\begin{array}{cc} K_{iJ}^{23} & =\int_{r_{1}}^{r_{2}}\left\{\frac{1}{2}\frac{\partial w_{0}}{\partial r}\left(A_{11}^{\left(1\right)}-c_{1}A_{11}^{\left(3\right)}\right)\frac{\partial \psi_{i}}{\partial r}\frac{\partial \phi_{J}}{\partial r}-c_{1}\left(A_{11}^{\left(4\right)}-c_{1}A_{11}^{\left(6\right)}\right)\left(\frac{\partial \psi_{i}}{\partial r}\frac{\partial^{2}\phi_{J}}{\partial r^{2}}+\frac{1}{r^{2}}\psi_{i}\frac{\partial \phi_{J}}{\partial r}\right)\right.\\ \; & \;+\frac{1}{2r}\frac{\partial w_{0}}{\partial r}\left(A_{12}^{\left(1\right)}-c_{1}A_{12}^{\left(3\right)}\right)\psi_{i}\frac{\partial \phi_{J}}{\partial r}-\frac{c_{1}}{r}\left(A_{12}^{\left(4\right)}-c_{1}A_{12}^{\left(6\right)}\right)\frac{\partial \psi_{i}}{\partial r}\frac{\partial \phi_{J}}{\partial r}\\ \; & \;-\frac{c_{1}}{r}\left(A_{12}^{\left(4\right)}-c_{1}A_{12}^{\left(6\right)}\right)\psi_{i}\frac{\partial^{2}\phi_{J}}{\partial r^{2}}+\left(A_{55}^{\left(0\right)}-3c_{2}A_{55}^{\left(2\right)}+c_{1}^{2}A_{55}^{\left(4\right)}\right)\psi_{i}\frac{\partial \phi_{J}}{\partial r}\\ \; & \;-\frac{1}{4}\left[\left(B^{\left(0\right)}-c_{2}^{2}B^{\left(4\right)}\right)\left(\frac{\partial \psi_{i}}{\partial r}\frac{\partial^{2}\phi_{J}}{\partial r^{2}}-\frac{1}{r}\frac{\partial \psi_{i}}{\partial r}\frac{\partial \phi_{J}}{\partial r}\right)-4c_{2}^{2}B^{\left(2\right)}\psi_{i}\frac{\partial \phi_{J}}{\partial r}\right.\\ \; & \;\left.\left.-\frac{1}{r}\left(B^{\left(0\right)}-c_{2}^{2}B^{\left(4\right)}\right)\left(\psi_{i}\frac{\partial^{2}\phi_{J}}{\partial r^{2}}-\frac{1}{r}\psi_{i}\frac{\partial \phi_{J}}{\partial r}\right)\right]\right\}rdr \end{array}$$

(A9) $$\begin{array}{cc} K_{iJ}^{24} & =\int_{r_{1}}^{r_{2}}\left\{-\frac{1}{2}\left(A_{11}^{\left(3\right)}-c_{1}A_{11}^{\left(5\right)}\right)\left(\frac{\partial \psi_{i}}{\partial r}\frac{\partial^{2}\phi_{J}}{\partial r^{2}}+\frac{1}{r^{2}}\psi_{i}\frac{\partial \phi_{J}}{\partial r}\right)+\frac{1}{r}\left(A_{12}^{\left(1\right)}-c_{1}A_{12}^{\left(3\right)}\right)\psi_{i}\phi_{J}\right.\\ \; & \;+\left(A_{12}^{\left(1\right)}-c_{1}A_{12}^{\left(3\right)}\right)\frac{\partial \psi_{i}}{\partial r}\phi_{J}-\frac{1}{2r}\left(A_{12}^{\left(3\right)}-c_{1}A_{12}^{\left(5\right)}\right)\left(\frac{\partial \psi_{i}}{\partial r}\frac{\partial \phi_{J}}{\partial r}+\psi_{i}\frac{\partial^{2}\phi_{J}}{\partial r^{2}}\right)\\ \; & \;+\frac{1}{2}\left[\frac{1}{r}\left(B^{\left(1\right)}-c_{2}B^{\left(3\right)}\right)\left(\frac{\partial \psi_{i}}{\partial r}\frac{\partial \phi_{J}}{\partial r}+\psi_{i}\frac{\partial^{2}\phi_{J}}{\partial r^{2}}-r\frac{\partial \psi_{i}}{\partial r}\frac{\partial^{2}\phi_{J}}{\partial r^{2}}\right)\right.\\ \; & \;\left.\left.-\frac{1}{r^{2}}\left(B^{\left(1\right)}-c_{2}B^{\left(3\right)}\right)\psi_{i}\frac{\partial \phi_{J}}{\partial r}+2c_{2}B^{\left(1\right)}\psi_{i}\frac{\partial \phi_{J}}{\partial r}\right]\right\}rdr \end{array}$$

(A10) $$\begin{array}{cc} K_{iJ}^{25} & =\int_{r_{1}}^{r_{2}}\left\{-\frac{1}{3}\left(A_{11}^{\left(4\right)}-c_{1}A_{11}^{\left(6\right)}\right)\left(\frac{\partial \psi_{i}}{\partial r}\frac{\partial^{2}\phi_{J}}{\partial r^{2}}+\frac{1}{r^{2}}\psi_{i}\frac{\partial \phi_{J}}{\partial r}\right)+\frac{2}{r}\left(A_{12}^{\left(2\right)}-c_{1}A_{12}^{\left(4\right)}\right)\psi_{i}\phi_{J}\right.\\ \; & \;+2\left(A_{12}^{\left(2\right)}-c_{1}A_{12}^{\left(4\right)}\right)\frac{\partial \psi_{i}}{\partial r}\phi_{J}-\frac{1}{3r}\left(A_{12}^{\left(4\right)}-c_{1}A_{12}^{\left(6\right)}\right)\left(\frac{\partial \psi_{i}}{\partial r}\frac{\partial \phi_{J}}{\partial r}+\psi_{i}\frac{\partial^{2}\phi_{J}}{\partial r^{2}}\right)\\ \; & \;+\frac{1}{2}\left[\frac{1}{r}\left(B^{\left(2\right)}-c_{2}B^{\left(4\right)}\right)\left(\frac{\partial \psi_{i}}{\partial r}\frac{\partial \phi_{J}}{\partial r}+\psi_{i}\frac{\partial^{2}\phi_{J}}{\partial r^{2}}-r\frac{\partial \psi_{i}}{\partial r}\frac{\partial^{2}\phi_{J}}{\partial r^{2}}\right)\right.\\ \; & \;\left.\left.-\frac{1}{r^{2}}\left(B^{\left(2\right)}-c_{2}B^{\left(4\right)}\right)\psi_{i}\frac{\partial \phi_{J}}{\partial r}+4c_{2}B^{\left(2\right)}\psi_{i}\frac{\partial \phi_{J}}{\partial r}\right]\right\}rdr \end{array}$$

(A11) $$\begin{array}{cc} K_{Ij}^{31} & =\left[A_{11}^{\left(0\right)}\left(\frac{\partial w_{0}}{\partial r}\right)\frac{\partial \phi_{I}}{\partial r}\frac{\partial \psi_{j}}{\partial r}-c_{1}A_{11}^{\left(3\right)}\left(\frac{\partial^{2}\phi_{I}}{\partial r^{2}}\frac{\partial \psi_{j}}{\partial r}+\frac{1}{r^{2}}\frac{\partial \phi_{I}}{\partial r}\psi_{j}\right)\right.\\ \; & \;\left.+\frac{A_{12}^{\left(0\right)}}{r}\left(\frac{\partial w_{0}}{\partial r}\right)\frac{\partial \phi_{I}}{\partial r}\psi_{j}-c_{1}\frac{A_{12}^{\left(3\right)}}{r}\left(\frac{\partial \phi_{I}}{\partial r}\frac{\partial \psi_{j}}{\partial r}+\frac{\partial^{2}\phi_{I}}{\partial r^{2}}\psi_{j}\right)\right]rdr \end{array}$$

(A12) $$\begin{array}{cc} K_{Ij}^{32} & =\int_{r_{1}}^{r_{2}}\left\{\frac{\partial w_{0}}{\partial r}\left(A_{11}^{\left(1\right)}-c_{1}A_{11}^{\left(3\right)}\right)\frac{\partial \phi_{I}}{\partial r}\frac{\partial \psi_{j}}{\partial r}-c_{1}\left(A_{11}^{\left(4\right)}-c_{1}A_{11}^{\left(6\right)}\right)\left(\frac{\partial^{2}\phi_{I}}{\partial r^{2}}\frac{\partial \psi_{j}}{\partial r}+\frac{1}{r^{2}}\frac{\partial \phi_{I}}{\partial r}\psi_{j}\right)\right.\\ \; & \;+\frac{1}{r}\frac{\partial w_{0}}{\partial r}\left(A_{12}^{\left(1\right)}-c_{1}A_{12}^{\left(3\right)}\right)\frac{\partial \phi_{I}}{\partial r}\psi_{i}-\frac{c_{1}}{r}\left(A_{12}^{\left(4\right)}-c_{1}A_{12}^{\left(6\right)}\right)\frac{\partial \phi_{I}}{\partial r}\frac{\partial \psi_{j}}{\partial r}\\ \; & \;-\frac{c_{1}}{r}\left(A_{12}^{\left(4\right)}-c_{1}A_{12}^{\left(6\right)}\right)\frac{\partial^{2}\phi_{I}}{\partial r^{2}}\psi_{j}+\left(A_{55}^{\left(0\right)}-3c_{2}A_{55}^{\left(2\right)}+c_{1}^{2}A_{55}^{\left(4\right)}\right)\frac{\partial \phi_{I}}{\partial r}\psi_{i}\\ \; & \;-\frac{1}{4}\left[\left(B^{\left(0\right)}-c_{2}^{2}B^{\left(4\right)}\right)\left(\frac{\partial^{2}\phi_{I}}{\partial r^{2}}\frac{\partial \psi_{j}}{\partial r}-\frac{1}{r}\frac{\partial \phi_{I}}{\partial r}\frac{\partial \psi_{j}}{\partial r}\right)-4c_{2}^{2}B^{\left(2\right)}\frac{\partial \phi_{I}}{\partial r}\psi_{j}\right.\\ \; & \;\left.\left.-\frac{1}{r}\left(B^{\left(0\right)}-c_{2}^{2}B^{\left(4\right)}\right)\left(\frac{\partial^{2}\phi_{I}}{\partial r^{2}}\psi_{j}-\frac{1}{r}\frac{\partial \phi_{I}}{\partial r}\psi_{j}\right)\right]\right\}rdr \end{array}$$

(A13) $$\begin{array}{cc} K_{IJ}^{33} & =\int_{r_{1}}^{r^{2}}\left\{\frac{1}{2}\left(\frac{\partial w_{0}}{\partial r}\right)\left(\frac{\partial w_{0}}{\partial r}A_{11}^{\left(0\right)}-\frac{c_{2}}{r}A_{12}^{\left(3\right)}\right)\frac{\partial \phi_{I}}{\partial r}\frac{\partial \phi_{J}}{\partial r}+\frac{c_{1}^{2}}{r}A_{12}^{\left(6\right)}\left(\frac{\partial^{2}\phi_{I}}{\partial r^{2}}\frac{\partial \phi_{J}}{\partial r}+\frac{\partial \phi_{I}}{\partial r}\frac{\partial^{2}\phi_{J}}{\partial r^{2}}\right)\right.\\ \; & \;-c_{1}A_{11}^{\left(3\right)}\left(\frac{\partial w_{0}}{\partial r}\right)\left(\frac{\partial \phi_{I}}{\partial r}\frac{\partial^{2}\phi_{J}}{\partial r^{2}}+\frac{1}{2}\frac{\partial^{2}\phi_{I}}{\partial r^{2}}\frac{\partial \phi_{J}}{\partial r}\right)+c_{1}^{2}A_{11}^{\left(6\right)}\left(\frac{\partial^{2}\phi_{I}}{\partial r^{2}}\frac{\partial^{2}\phi_{J}}{\partial r^{2}}+\frac{1}{r^{2}}\frac{\partial \phi_{I}}{\partial r}\frac{\partial \phi_{J}}{\partial r}\right)\\ \; & \;+\left(A_{55}^{\left(0\right)}-3c_{2}A_{55}^{\left(2\right)}+c_{2}^{2}A_{55}^{\left(4\right)}\right)\frac{\partial \phi_{I}}{\partial r}\frac{\partial \phi_{J}}{\partial r}+\frac{1}{4}\left[c2^{2}B^{\left(4\right)}\left(\frac{\partial^{2}\phi_{I}}{\partial r^{2}}\frac{\partial^{2}\phi_{J}}{\partial r^{2}}+\frac{1}{r^{2}}\frac{\partial \phi_{I}}{\partial r}\frac{\partial \phi_{J}}{\partial r}\right)\right.\\ \; & \;+\left(B^{\left(0\right)}+2c_{2}B^{\left(2\right)}\right)\left(\frac{\partial^{2}\phi_{I}}{\partial r^{2}}\frac{\partial^{2}\phi_{J}}{\partial r^{2}}+\frac{1}{r}\frac{\partial \phi_{I}}{\partial r}\frac{\partial \phi_{J}}{\partial r}\right)\\ \; & \;\left.\left.-\frac{1}{r}\left(B^{\left(0\right)}+2c_{2}B^{\left(2\right)}+c_{2}^{2}\right)\left(\frac{\partial^{2}\phi_{I}}{\partial r^{2}}\frac{\partial \phi_{J}}{\partial r}+\frac{\partial \phi_{I}}{\partial r}\frac{\partial^{2}\phi_{J}}{\partial r^{2}}\right)\right]\right\}rdr \end{array}$$

(A14) $$\begin{array}{cc} K_{IJ}^{34} & =\int_{r_{1}}^{r^{2}}\left\{-\frac{1}{2}\left(\frac{\partial w_{0}}{\partial r}\right)\left(A_{11}^{\left(2\right)}\frac{\partial \phi_{I}}{\partial r}\frac{\partial^{2}\phi_{J}}{\partial r^{2}}+\frac{A_{12}^{\left(2\right)}}{r}\frac{\partial \phi_{I}}{\partial r}\frac{\partial \phi_{J}}{\partial r}-2A_{12}^{\left(0\right)}\frac{\partial \phi_{I}}{\partial r}\phi_{J}\right)\right.\\ \; & \;+\frac{c_{1}}{2}A_{11}^{\left(5\right)}\left(\frac{\partial^{2}\phi_{I}}{\partial r^{2}}\frac{\partial^{2}\phi_{J}}{\partial r^{2}}+\frac{1}{r^{2}}\frac{\partial \phi_{I}}{\partial r}\frac{\partial \phi_{J}}{\partial r}\right)-c_{1}A_{12}^{\left(3\right)}\left(\frac{1}{r}\frac{\partial \phi_{I}}{\partial r}\phi_{J}+\frac{\partial^{2}\phi_{I}}{\partial r^{2}}\phi_{J}\right)\\ \; & \;+\frac{c_{1}}{2r}A_{12}^{\left(5\right)}\left(\frac{\partial \phi_{I}}{\partial r}\frac{\partial^{2}\phi_{J}}{\partial r^{2}}+\frac{\partial^{2}\phi_{I}}{\partial r^{2}}\frac{\partial \phi_{J}}{\partial r}\right)+\frac{1}{2}\left[2c_{2}B^{\left(1\right)}\frac{\partial \phi_{I}}{\partial r}\frac{\partial \phi_{J}}{\partial r}\right.\\ \; & \;\left.\left.+\left(B^{\left(1\right)}+c_{2}B^{\left(3\right)}\right)\left(\frac{\partial^{2}\phi_{I}}{\partial r^{2}}\frac{\partial^{2}\phi_{J}}{\partial r^{2}}-\frac{1}{r}\frac{\partial^{2}\phi_{I}}{\partial r^{2}}\frac{\partial \phi_{J}}{\partial r}-\frac{1}{r}\frac{\partial \phi_{I}}{\partial r}\frac{\partial^{2}\phi_{J}}{\partial r^{2}}+\frac{1}{r^{2}}\frac{\partial \phi_{I}}{\partial r}\frac{\partial \phi_{J}}{\partial r}\right)\right]\right\}rdr \end{array}$$

(A15) $$\begin{array}{cc} K_{IJ}^{35} & =\int_{r_{1}}^{r^{2}}\left\{-\frac{1}{3}\left(\frac{\partial w_{0}}{\partial r}\right)\left(A_{11}^{\left(3\right)}\frac{\partial \phi_{I}}{\partial r}\frac{\partial^{2}\phi_{J}}{\partial r^{2}}+\frac{A_{12}^{\left(3\right)}}{r}\frac{\partial \phi_{I}}{\partial r}\frac{\partial \phi_{J}}{\partial r}-6A_{12}^{\left(1\right)}\frac{\partial \phi_{I}}{\partial r}\phi_{J}\right)\right.\\ \; & \;+\frac{c_{1}}{3}A_{11}^{\left(6\right)}\left(\frac{\partial^{2}\phi_{I}}{\partial r^{2}}\frac{\partial^{2}\phi_{J}}{\partial r^{2}}+\frac{1}{r^{2}}\frac{\partial \phi_{I}}{\partial r}\frac{\partial \phi_{J}}{\partial r}\right)-2c_{1}A_{12}^{\left(4\right)}\left(\frac{1}{r}\frac{\partial \phi_{I}}{\partial r}\phi_{J}+\frac{\partial^{2}\phi_{I}}{\partial r^{2}}\phi_{J}\right)\\ \; & \;-\frac{c_{1}}{3r}A_{12}^{\left(6\right)}\left(\frac{\partial \phi_{I}}{\partial r}\frac{\partial^{2}\phi_{J}}{\partial r^{2}}+\frac{\partial^{2}\phi_{I}}{\partial r^{2}}\frac{\partial \phi_{J}}{\partial r}\right)+\frac{1}{2}\left[4c_{2}B^{\left(2\right)}\frac{\partial \phi_{I}}{\partial r}\frac{\partial \phi_{J}}{\partial r}\right.\\ \; & \;\left.\left.+\left(B^{\left(2\right)}+c_{2}B^{\left(4\right)}\right)\left(\frac{\partial^{2}\phi_{I}}{\partial r^{2}}\frac{\partial^{2}\phi_{J}}{\partial r^{2}}-\frac{1}{r}\frac{\partial^{2}\phi_{I}}{\partial r^{2}}\frac{\partial \phi_{J}}{\partial r}-\frac{1}{r}\frac{\partial \phi_{I}}{\partial r}\frac{\partial^{2}\phi_{J}}{\partial r^{2}}+\frac{1}{r^{2}}\frac{\partial \phi_{I}}{\partial r}\frac{\partial \phi_{J}}{\partial r}\right)\right]\right\}rdr \end{array}$$

(A16) $$\begin{array}{cc} K_{Ij}^{41} & =\int_{r_{1}}^{r_{2}}\left[-\frac{A_{11}^{\left(2\right)}}{2}\left(\frac{\partial^{2}\phi_{i}}{\partial r^{2}}\frac{\partial \psi_{j}}{\partial r}+\frac{1}{r^{2}}\frac{\partial \phi_{I}}{\partial r}\psi_{j}\right)+A_{12}^{\left(0\right)}\left(\phi_{I}\frac{\partial \psi_{j}}{\partial r}+\frac{1}{r}\phi_{I}\psi_{j}\right)\right.\\ \; & \;\left.-\frac{A_{12}^{\left(2\right)}}{2r}\left(\frac{\partial \phi_{I}}{\partial r}\frac{\partial \psi_{j}}{\partial r}+\frac{\partial^{2}\phi_{I}}{\partial r^{2}}\psi_{i}\right)\right]rdr \end{array}$$

(A17) $$\begin{array}{cc} K_{IJ}^{42} & =\int_{r_{1}}^{r_{2}}\left\{-\frac{1}{2}\left(A_{11}^{\left(3\right)}-c_{1}A_{11}^{\left(5\right)}\right)\left(\frac{\partial^{2}\phi_{I}}{\partial r^{2}}\frac{\partial \psi_{j}}{\partial r}+\frac{1}{r^{2}}\frac{\partial \phi_{I}}{\partial r}\psi_{j}\right)+\frac{1}{r}\left(A_{12}^{\left(1\right)}-c_{1}A_{12}^{\left(3\right)}\right)\phi_{I}\psi_{i}\right.\\ \; & \;+\left(A_{12}^{\left(1\right)}-c_{1}A_{12}^{\left(3\right)}\right)\frac{\phi_{I}\partial \psi_{j}}{\partial r}-\frac{1}{2r}\left(A_{12}^{\left(3\right)}-c_{1}A_{12}^{\left(5\right)}\right)\left(\frac{\partial \psi_{i}}{\partial r}\frac{\partial \phi_{J}}{\partial r}+\frac{\partial^{2}\phi_{I}}{\partial r^{2}}\psi_{j}\right)\\ \; & \;+\frac{1}{2}\left[\frac{1}{r}\left(B^{\left(1\right)}-c_{2}B^{\left(3\right)}\right)\left(\frac{\partial \phi_{I}}{\partial r}\frac{\partial \psi_{j}}{\partial r}+\frac{\partial^{2}\phi_{I}}{\partial r^{2}}\psi_{j}-r\frac{\partial^{2}\phi_{I}}{\partial r^{2}}\frac{\partial \psi_{j}}{\partial r}\right)\right.\\ \; & \;\left.\left.-\frac{1}{r^{2}}\left(B^{\left(1\right)}-c_{2}B^{\left(3\right)}\right)\frac{\partial \phi_{I}}{\partial r}\psi_{j}+2c_{2}B^{\left(1\right)}\frac{\partial \phi_{I}}{\partial r}\psi_{j}\right]\right\}rdr \end{array}$$

(A18) $$\begin{array}{cc} K_{IJ}^{43} & =\int_{r_{1}}^{r^{2}}\left\{-\frac{1}{4}\left(\frac{\partial w_{0}}{\partial r}\right)\left(A_{11}^{\left(2\right)}\frac{\partial \phi_{I}}{\partial r}\frac{\partial^{2}\phi_{J}}{\partial r^{2}}+\frac{A_{12}^{\left(2\right)}}{r}\frac{\partial \phi_{I}}{\partial r}\frac{\partial \phi_{J}}{\partial r}-2A_{12}^{\left(0\right)}\frac{\partial \phi_{I}}{\partial r}\phi_{J}\right)\right.\\ \; & \;+\frac{c_{1}}{2}A_{11}^{\left(5\right)}\left(\frac{\partial^{2}\phi_{I}}{\partial r^{2}}\frac{\partial^{2}\phi_{J}}{\partial r^{2}}+\frac{1}{r^{2}}\frac{\partial \phi_{I}}{\partial r}\frac{\partial \phi_{J}}{\partial r}\right)-c_{1}A_{12}^{\left(3\right)}\left(\frac{1}{r}\phi_{I}\frac{\partial \phi_{J}}{\partial r}+\phi_{I}\frac{\partial^{2}\phi_{J}}{\partial r^{2}}\right)\\ \; & \;+\frac{c_{1}}{2r}A_{12}^{\left(5\right)}\left(\frac{\partial^{2}\phi_{I}}{\partial r^{2}}\frac{\partial \phi_{J}}{\partial r}+\frac{\partial \phi_{I}}{\partial r}\frac{\partial^{2}\phi_{J}}{\partial r^{2}}\right)+\frac{1}{2}\left[2c_{2}B^{\left(1\right)}\frac{\partial \phi_{I}}{\partial r}\frac{\partial \phi_{J}}{\partial r}\right.\\ \; & \;\left.\left.+\left(B^{\left(1\right)}+c_{2}B^{\left(3\right)}\right)\left(\frac{\partial^{2}\phi_{I}}{\partial r^{2}}\frac{\partial^{2}\phi_{J}}{\partial r^{2}}-\frac{1}{r}\frac{\partial \phi_{I}}{\partial r}\frac{\partial^{2}\phi_{J}}{\partial r^{2}}-\frac{1}{r}\frac{\partial^{2}\phi_{I}}{\partial r^{2}}\frac{\partial \phi_{J}}{\partial r}+\frac{1}{r^{2}}\frac{\partial \phi_{I}}{\partial r}\frac{\partial \phi_{J}}{\partial r}\right)\right]\right\}rdr \end{array}$$

(A19) $$\begin{array}{cc} K_{IJ}^{44} & =\int_{r_{1}}^{r^{2}}\left\{A_{11}^{\left(0\right)}\phi_{I}\phi_{J}+\frac{A_{11}^{\left(4\right)}}{4}\left(\frac{\partial^{2}\phi_{I}}{\partial r^{2}}\frac{\partial^{2}\phi_{J}}{\partial r^{2}}+\frac{1}{r^{2}}\frac{\partial \phi_{I}}{\partial r}\frac{\partial \phi_{J}}{\partial r}\right)-\frac{A_{12}^{\left(2\right)}}{2}\left(\frac{\partial^{2}\phi_{I}}{\partial r^{2}}\phi_{J}+\phi_{I}\frac{\partial^{2}\phi_{J}}{\partial r^{2}}\right)\right.\\ \; & \;-\frac{A_{12}^{\left(2\right)}}{2r}\left(\frac{\partial \phi_{I}}{\partial r}\phi_{J}+\phi_{I}\frac{\partial \phi_{J}}{\partial r}\right)+\frac{A_{12}^{\left(4\right)}}{4r}\left(\frac{\partial^{2}\phi_{I}}{\partial r^{2}}\frac{\partial \phi_{J}}{\partial r}+\frac{\partial \phi_{I}}{\partial r}\frac{\partial^{2}\phi_{J}}{\partial r^{2}}\right)+B^{\left(0\right)}\frac{\partial \phi_{I}}{\partial r}\frac{\partial \phi_{J}}{\partial r}\\ \; & \;\left.+B^{\left(2\right)}\left[\frac{\partial^{2}\phi_{I}}{\partial r^{2}}\frac{\partial^{2}\phi_{J}}{\partial r^{2}}-\frac{1}{r}\left(\frac{\partial^{2}\phi_{I}}{\partial r^{2}}\frac{\partial \phi_{J}}{\partial r}+\frac{\partial \phi_{I}}{\partial r}\frac{\partial^{2}\phi_{J}}{\partial r^{2}}\right)+\frac{1}{r^{2}}\frac{\partial \phi_{I}}{\partial r}\frac{\partial \phi_{J}}{\partial r}\right]\right\}rdr \end{array}$$

(A20) $$\begin{array}{cc} K_{IJ}^{45} & =\int_{r_{1}}^{r^{2}}\left\{A_{11}^{\left(1\right)}\phi_{I}\phi_{J}+\frac{A_{11}^{\left(5\right)}}{6}\left(\frac{\partial^{2}\phi_{I}}{\partial r^{2}}\frac{\partial^{2}\phi_{J}}{\partial r^{2}}+\frac{1}{r^{2}}\frac{\partial \phi_{I}}{\partial r}\frac{\partial \phi_{J}}{\partial r}\right)-\frac{A_{12}^{\left(3\right)}}{3}\left(3\frac{\partial^{2}\phi_{I}}{\partial r^{2}}\phi_{J}+\phi_{I}\frac{\partial^{2}\phi_{J}}{\partial r^{2}}\right)\right.\\ \; & \;-\frac{A_{12}^{\left(3\right)}}{3r}\left(3\frac{\partial \phi_{I}}{\partial r}\phi_{J}+\phi_{I}\frac{\partial \phi_{J}}{\partial r}\right)+\frac{A_{12}^{\left(5\right)}}{6r}\left(\frac{\partial^{2}\phi_{I}}{\partial r^{2}}\frac{\partial \phi_{J}}{\partial r}+\frac{\partial \phi_{I}}{\partial r}\frac{\partial^{2}\phi_{J}}{\partial r^{2}}\right)+2B^{\left(1\right)}\frac{\partial \phi_{I}}{\partial r}\frac{\partial \phi_{J}}{\partial r}\\ \; & \;\left.+B^{\left(3\right)}\left[\frac{\partial^{2}\phi_{I}}{\partial r^{2}}\frac{\partial^{2}\phi_{J}}{\partial r^{2}}-\frac{1}{r}\left(\frac{\partial^{2}\phi_{I}}{\partial r^{2}}\frac{\partial \phi_{J}}{\partial r}+\frac{\partial \phi_{I}}{\partial r}\frac{\partial^{2}\phi_{J}}{\partial r^{2}}\right)+\frac{1}{r^{2}}\frac{\partial \phi_{I}}{\partial r}\frac{\partial \phi_{J}}{\partial r}\right]\right\}rdr \end{array}$$

(A21) $$\begin{array}{cc} K_{Ij}^{51} & =\int_{r_{1}}^{r_{2}}\left[-\frac{A_{11}^{\left(3\right)}}{3}\left(\frac{\partial^{2}\phi_{I}}{\partial r^{2}}\frac{\partial \psi_{j}}{\partial r}+\frac{1}{r^{2}}\frac{\partial \phi_{I}}{\partial r}\psi_{j}\right)+2A_{12}^{\left(1\right)}c_{2}\left(\phi_{I}\frac{\partial \psi_{j}}{\partial r}+\frac{1}{r}\phi_{I}\psi_{j}\right)\right.\\ \; & \;\left.-\frac{A_{12}^{\left(3\right)}}{3r}\left(\frac{\partial \phi_{I}}{\partial r}\frac{\partial \psi_{j}}{\partial r}+\frac{\partial^{2}\phi_{I}}{\partial r^{2}}\psi_{j}\right)\right]rdr \end{array}$$

(A22) $$\begin{array}{cc} K_{Ij}^{52} & =\int_{r_{1}}^{r_{2}}\left\{-\frac{1}{3}\left(A_{11}^{\left(4\right)}-c_{1}A_{11}^{\left(6\right)}\right)\left(\frac{\partial^{2}\phi_{I}}{\partial r^{2}}\frac{\partial \psi_{j}}{\partial r}+\frac{1}{r^{2}}\frac{\partial \phi_{I}}{\partial r}\psi_{i}\right)+\frac{2}{r}\left(A_{12}^{\left(2\right)}-c_{1}A_{12}^{\left(4\right)}\right)\phi_{I}\psi_{j}\right.\\ \; & \;+2\left(A_{12}^{\left(2\right)}-c_{1}A_{12}^{\left(4\right)}\right)\phi_{I}\frac{\partial \psi_{j}}{\partial r}-\frac{1}{3r}\left(A_{12}^{\left(4\right)}-c_{1}A_{12}^{\left(6\right)}\right)\left(\frac{\partial \phi_{I}}{\partial r}\frac{\partial \psi_{j}}{\partial r}+\frac{\partial^{2}\phi_{I}}{\partial r^{2}}\psi_{j}\right)\\ \; & \;+\frac{1}{2}\left[\frac{1}{r}\left(B^{\left(2\right)}-c_{2}B^{\left(4\right)}\right)\left(\frac{\partial \phi_{I}}{\partial r}\frac{\partial \psi_{j}}{\partial r}+\frac{\partial^{2}\phi_{I}}{\partial r^{2}}\psi_{j}-r\frac{\partial^{2}\phi_{I}}{\partial r^{2}}\frac{\partial \psi_{j}}{\partial r}\right)\right.\\ \; & \;\left.\left.-\frac{1}{r^{2}}\left(B^{\left(2\right)}-c_{2}B^{\left(4\right)}\right)\frac{\partial \phi_{I}}{\partial r}\psi_{j}+4c_{2}B^{\left(2\right)}\frac{\partial \phi_{I}}{\partial r}\psi_{j}\right]\right\}rdr \end{array}$$

(A23) $$\begin{array}{cc} K_{IJ}^{53} & =\int_{r_{1}}^{r^{2}}\left\{-\frac{1}{6}\left(\frac{\partial w_{0}}{\partial r}\right)\left(A_{11}^{\left(3\right)}\frac{\partial^{2}\phi_{I}}{\partial r^{2}}\frac{\partial \phi_{J}}{\partial r}+\frac{A_{12}^{\left(3\right)}}{r}\frac{\partial \phi_{I}}{\partial r}\frac{\partial \phi_{J}}{\partial r}-6A_{12}^{\left(1\right)}\phi_{I}\frac{\partial \phi_{J}}{\partial r}\right)\right.\\ \; & \;+\frac{c_{1}}{3}A_{11}^{\left(6\right)}\left(\frac{\partial^{2}\phi_{I}}{\partial r^{2}}\frac{\partial^{2}\phi_{J}}{\partial r^{2}}+\frac{1}{r^{2}}\frac{\partial \phi_{I}}{\partial r}\frac{\partial \phi_{J}}{\partial r}\right)-2c_{1}A_{12}^{\left(4\right)}\left(\frac{1}{r}\phi_{I}\frac{\partial \phi_{J}}{\partial r}+\phi_{I}\frac{\partial^{2}\phi_{J}}{\partial r^{2}}\right)\\ \; & \;-\frac{c_{1}}{3r}A_{12}^{\left(6\right)}\left(\frac{\partial^{2}\phi_{I}}{\partial r^{2}}\frac{\partial \phi_{J}}{\partial r}+\frac{\partial \phi_{I}}{\partial r}\frac{\partial^{2}\phi_{J}}{\partial r^{2}}\right)+\frac{1}{2}\left[4c_{2}B^{\left(2\right)}\frac{\partial \phi_{I}}{\partial r}\frac{\partial \phi_{J}}{\partial r}\right.\\ \; & \;\left.\left.+\left(B^{\left(2\right)}+c_{2}B^{\left(4\right)}\right)\left(\frac{\partial^{2}\phi_{I}}{\partial r^{2}}\frac{\partial^{2}\phi_{J}}{\partial r^{2}}-\frac{1}{r}\frac{\partial \phi_{I}}{\partial r}\frac{\partial^{2}\phi_{J}}{\partial r^{2}}-\frac{1}{r}\frac{\partial^{2}\phi_{I}}{\partial r^{2}}\frac{\partial \phi_{J}}{\partial r}+\frac{1}{r^{2}}\frac{\partial \phi_{I}}{\partial r}\frac{\partial \phi_{J}}{\partial r}\right)\right]\right\}rdr \end{array}$$

(A24) $$\begin{array}{cc} K_{IJ}^{54} & =\int_{r_{1}}^{r^{2}}\left\{A_{11}^{\left(1\right)}\phi_{I}\phi_{J}+\frac{A_{11}^{\left(5\right)}}{6}\left(\frac{\partial^{2}\phi_{I}}{\partial r^{2}}\frac{\partial^{2}\phi_{J}}{\partial r^{2}}+\frac{1}{r^{2}}\frac{\partial \phi_{I}}{\partial r}\frac{\partial \phi_{J}}{\partial r}\right)-\frac{A_{12}^{\left(3\right)}}{3}\left(3\phi_{I}\frac{\partial^{2}\phi_{J}}{\partial r^{2}}+\frac{\partial^{2}\phi_{I}}{\partial r^{2}}\phi_{J}\right)\right.\\ \; & \;-\frac{A_{12}^{\left(3\right)}}{3r}\left(3\phi_{I}\frac{\partial \phi_{J}}{\partial r}+\frac{\partial \phi_{I}}{\partial r}\phi_{J}\right)+\frac{A_{12}^{\left(5\right)}}{6r}\left(\frac{\partial \phi_{I}}{\partial r}\frac{\partial^{2}\phi_{J}}{\partial r^{2}}+\frac{\partial^{2}\phi_{I}}{\partial r^{2}}\frac{\partial \phi_{J}}{\partial r}\right)+2B^{\left(1\right)}\frac{\partial \phi_{I}}{\partial r}\frac{\partial \phi_{J}}{\partial r}\\ \; & \;\left.+B^{\left(3\right)}\left[\frac{\partial^{2}\phi_{I}}{\partial r^{2}}\frac{\partial^{2}\phi_{J}}{\partial r^{2}}-\frac{1}{r}\left(\frac{\partial \phi_{I}}{\partial r}\frac{\partial^{2}\phi_{J}}{\partial r^{2}}+\frac{\partial^{2}\phi_{I}}{\partial r^{2}}\frac{\partial \phi_{J}}{\partial r}\right)+\frac{1}{r^{2}}\frac{\partial \phi_{I}}{\partial r}\frac{\partial \phi_{J}}{\partial r}\right]\right\}rdr \end{array}$$

(A25) $$\begin{array}{cc} K_{IJ}^{55} & =\int_{r_{1}}^{r^{2}}\left\{4A_{11}^{\left(2\right)}\phi_{I}\phi_{J}+\frac{A_{11}^{\left(6\right)}}{9}\left(\frac{\partial^{2}\phi_{I}}{\partial r^{2}}\frac{\partial^{2}\phi_{J}}{\partial r^{2}}+\frac{1}{r^{2}}\frac{\partial \phi_{I}}{\partial r}\frac{\partial \phi_{J}}{\partial r}\right)-\frac{2A_{12}^{\left(4\right)}}{3}\left(\frac{\partial^{2}\phi_{I}}{\partial r^{2}}\phi_{J}+\phi_{I}\frac{\partial^{2}\phi_{J}}{\partial r^{2}}\right)\right.\\ \; & \;-\frac{2A_{12}^{\left(4\right)}}{3r}\left(\frac{\partial \phi_{I}}{\partial r}\phi_{J}+\phi_{I}\frac{\partial \phi_{J}}{\partial r}\right)+\frac{A_{12}^{\left(6\right)}}{9r}\left(\frac{\partial^{2}\phi_{I}}{\partial r^{2}}\frac{\partial \phi_{J}}{\partial r}+\frac{\partial \phi_{I}}{\partial r}\frac{\partial^{2}\phi_{J}}{\partial r^{2}}\right)+4B^{\left(2\right)}\frac{\partial \phi_{I}}{\partial r}\frac{\partial \phi_{J}}{\partial r}\\ \; & \;\left.+B^{\left(4\right)}\left[\frac{\partial^{2}\phi_{I}}{\partial r^{2}}\frac{\partial^{2}\phi_{J}}{\partial r^{2}}-\frac{1}{r}\left(\frac{\partial^{2}\phi_{I}}{\partial r^{2}}\frac{\partial \phi_{J}}{\partial r}+\frac{\partial \phi_{I}}{\partial r}\frac{\partial^{2}\phi_{J}}{\partial r^{2}}\right)+\frac{1}{r^{2}}\frac{\partial \phi_{I}}{\partial r}\frac{\partial \phi_{J}}{\partial r}\right]\right\}rdr \end{array}$$

(A26) $$\begin{array}{cc} F_{i}^{1} & =\int_{r_{1}}^{r^{2}}\left\{\left(f_{r}^{\left(0\right)}+q_{r}^{\left(0\right)}\right)\psi_{i}+T_{r}^{\left(0\right)}\frac{\partial \psi_{i}}{\partial r}+\frac{1}{r}T_{\theta }^{\left(0\right)}\psi_{i}\right\}\;rdr \end{array}$$

(A27) $$\begin{array}{cc} F_{i}^{2} & =\int_{r_{1}}^{r^{2}}\left\{\left[\left(f_{r}^{\left(1\right)}+q_{r}^{\left(1\right)}\right)-c_{1}\left(f_{r}^{\left(3\right)}+q_{r}^{\left(3\right)}\right)+\left(c_{\theta }^{\left(0\right)}+p_{\theta }^{\left(0\right)}\right)-c_{2}\left(c_{\theta }^{\left(2\right)}+p_{\theta }^{\left(2\right)}\right)\right]\psi_{i}\right.\\ \; & \;\left.+\left(T_{r}^{\left(1\right)}-c_{1}T_{r}^{\left(3\right)}\right)\frac{\partial \psi_{i}}{\partial r}+\frac{1}{r}\left(T_{\theta }^{\left(1\right)}-c_{1}T_{\theta }^{\left(3\right)}\right)\psi_{i}\right\}\;rdr \end{array}$$

(A28) $$\begin{array}{cc} F_{I}^{3} & =\int_{r_{1}}^{r^{2}}\left\{\left(f_{z}^{\left(0\right)}+q_{z}^{\left(0\right)}\right)\phi_{I}-\left[c_{1}\left(f_{r}^{\left(3\right)}+q_{r}^{\left(3\right)}\right)+\left(c_{\theta }^{\left(0\right)}+p_{\theta }^{\left(0\right)}\right)+c_{2}\left(c_{\theta }^{\left(2\right)}+p_{\theta }^{\left(2\right)}\right)\right]\frac{\partial \phi_{I}}{\partial r}\right.\\ \; & \;\left.+\left(\frac{\partial w_{0}}{\partial r}\right)T_{r}^{\left(0\right)}\frac{\partial \phi_{I}}{\partial r}-c_{1}T_{r}^{\left(3\right)}\frac{\partial^{2}\phi_{I}}{\partial r^{2}}+\frac{1}{r}T_{\theta }^{\left(3\right)}\frac{\partial \phi_{I}}{\partial r}\right\}\;rdr \end{array}$$

(A29) $$\begin{array}{cc} F_{I}^{4} & =\int_{r_{1}}^{r^{2}}\left\{\left(f_{z}^{\left(1\right)}+q_{z}^{\left(1\right)}\right)\phi_{I}-\left[\frac{1}{2}\left(f_{r}^{\left(2\right)}+q_{r}^{\left(2\right)}\right)+2\left(c_{\theta }^{\left(1\right)}+p_{\theta }^{\left(1\right)}\right)\right]\frac{\partial \phi_{I}}{\partial r}\right.\\ \; & \;\left.+T_{z}^{\left(0\right)}\phi_{I}-\frac{1}{2}T_{r}^{\left(2\right)}\frac{\partial^{2}\phi_{I}}{\partial r^{2}}-\frac{1}{2r}T_{\theta }^{\left(2\right)}\frac{\partial \phi_{I}}{\partial r}\right\}\;rdr \end{array}$$

(A30) $$\begin{array}{cc} F_{I}^{5} & =\int_{r_{1}}^{r^{2}}\left\{\left(f_{z}^{\left(2\right)}+q_{z}^{\left(2\right)}\right)\phi_{I}-\left[\frac{1}{3}\left(f_{r}^{\left(3\right)}+q_{r}^{\left(3\right)}\right)+3\left(c_{\theta }^{\left(2\right)}+p_{\theta }^{\left(2\right)}\right)\right]\frac{\partial \phi_{I}}{\partial r}\right.\\ \; & \;\left.+2T_{z}^{\left(1\right)}\phi_{I}-\frac{1}{3}T_{r}^{\left(3\right)}\frac{\partial^{2}\phi_{I}}{\partial r^{2}}-\frac{1}{3r}T_{\theta }^{\left(3\right)}\frac{\partial \phi_{I}}{\partial r}\right\}\;rdr \end{array}$$

where

(A31) $$\begin{array}{cc} A_{11}^{\left(k\right)} & =\int_{-\frac{h}{2}}^{\frac{h}{2}}{\left(z\right)}^{k}\;\left(1-\nu \right)\Lambda \left(z\right)\;dz,\;A_{12}^{\left(k\right)}=\int_{-\frac{h}{2}}^{\frac{h}{2}}{\left(z\right)}^{k}\;\nu \Lambda \left(z\right)\;dz,\\ A_{55}^{\left(k\right)} & =\int_{-\frac{h}{2}}^{\frac{h}{2}}{\left(z\right)}^{k}\;\frac{\left(1-2\nu \right)}{2}\Lambda \left(z\right)\;dz,\;B^{\left(k\right)}=\int_{-\frac{h}{2}}^{\frac{h}{2}}{\left(z\right)}^{k}\;\ell^{2}\left(1-2\nu \right)\Lambda \left(z\right)\;dz,\\ f_{\xi }^{\left(k\right)} & =\int_{-\frac{h}{2}}^{\frac{h}{2}}{\left(z\right)}^{i}\;{\bar{f}}_{\xi }\;dz,\;q_{\xi }^{\left(k\right)}={\left(\frac{h}{2}\right)}^{\left(k\right)}\left[q_{\xi }^{t}+{\left(-1\right)}^{\left(k\right)}q_{\xi }^{b}\right],\\ {\hat{c}}_{\theta }^{\left(k\right)} & =\int_{-\frac{h}{2}}^{\frac{h}{2}}{\left(z\right)}^{k}\;{\bar{c}}_{\theta }\;dz,\;p_{\theta }^{\left(k\right)}={\left(\frac{h}{2}\right)}^{\left(k\right)}\left[p_{\theta }^{t}+{\left(-1\right)}^{\left(k\right)}p_{\theta }^{b}\right],\\ T_{\xi }^{\left(k\right)} & =\int_{-\frac{h}{2}}^{\frac{h}{2}}{\left(z\right)}^{k}\sigma_{ij}^{T}\;dz \end{array}$$

for k=0,1,2,3,4,6, ξ=r,θ,z, and $\sigma_{ij}^{T}$ is the thermal stress due to the temperature difference $\left(T-T_{0}\right)$.
